# The avian egg exhibits general allometric invariances in mechanical design

**DOI:** 10.1038/s41598-017-14552-0

**Published:** 2017-10-27

**Authors:** Jia-Yang Juang, Pin-Yi Chen, Da-Chang Yang, Shang-Ping Wu, An Yen, Hsin-I. Hsieh

**Affiliations:** 1 Department of Mechanical Engineering, National Taiwan University, Taipei, 10617 Taiwan; 2 Taipei Zoo, Taipei, 11656 Taiwan

## Abstract

The avian egg exhibits extraordinary diversity in size, shape and color, and has a key role in avian adaptive radiations. Despite extensive work, our understanding of the underlying principles that guide the “design” of the egg as a load-bearing structure remains incomplete, especially over broad taxonomic scales. Here we define a dimensionless number *C*, a function of egg weight, stiffness and dimensions, to quantify how stiff an egg is with respect to its weight after removing geometry-induced rigidity. We analyze eggs of 463 bird species in 36 orders across five orders of magnitude in body mass, and find that C number is nearly invariant for most species, including tiny hummingbirds and giant elephant birds. This invariance or “design guideline” dictates that evolutionary changes in shell thickness and Young’s modulus, both contributing to shell stiffness, are constrained by changes in egg weight. Our analysis illuminates unique reproductive strategies of brood parasites, kiwis, and megapodes, and quantifies the loss of safety margin for contact incubation due to artificial selection and environmental toxins. Our approach provides a mechanistic framework for a better understanding of the mechanical design of the avian egg, and may provide clues to the evolutionary origin of contact incubation of amniote eggs.

## Introduction

Birds comprise over 10,000 living species^1^, and exhibit an extraordinary diversity in morphology, behavior^2,3^, and lifestyle traits such as diet, developmental mode, breeding system and nest type^4^. Reproduction through eggs has been a very successful system for birds to spread and flourish in every terrestrial habitat^5–7^. The eggshell protects the developing embryo from damage and excessive water loss, and provides the embryo with calcium for its skeleton^5^. Most birds incubate their eggs, except for megapodes and brood parasites (incubated by their hosts). During contact incubation, the egg is subjected to the weight of the incubating bird and possible impact as eggs are moved around in the nest by the bird, known as “egg turning”^5^.

The mechanical design of a load-bearing structure is often examined from three standpoints: stiffness, strength and stability, which relate respectively to its ability to resist deflection, to resist permanent deformation or fracture, and to retain its equilibrium shape^8^. As a load-bearing structure, the eggshell must be sufficiently stiff to resist deformation, be sufficiently strong to resist fracture, and be stable to avoid buckling. Meanwhile, it has to be breakable from the inside for the hatchling^9^. Those are design trade-offs that must be adequately balanced.

Despite extensive work on the allometric scaling of egg properties^5,10^, it remains unclear why such empirical relations exist. The egg size varies dramatically across bird species. A tiny egg of the 3-g vervain hummingbird (*Mellisuga minima*) weighed less than 0.3 g, and a giant egg of the extinct 450-kg elephant bird (*Aepyornis maximus*) weighed over 9,000 g. Also, avian eggs exhibit a variety of shapes^11^: spherical in owls, elliptical in hummingbirds, conical in shorebirds, and other forms with different degrees of asymmetry and ellipticity^12^.

The stiffness of nonspherical shells is determined by (i) the thickness *t*, (ii) the local curvature *κ* of the loading point, or equivalently egg size and shape, (iii) the material properties (Young’s modulus *E*), and (iv) the in-out differential pressure (negligible in freshly laid eggs^13^). Recent work^14,15^ showed that the rigidity at the pole of an ellipsoidal shell scales proportional to the aspect ratio *B*/*A*, known as geometry-induced rigidity (GIR), defined as the amount by which a nonspherical shell is stiffened when compared to a spherical shell with the same thickness and material properties^14^. For example, for a given material and thickness, an ellipsoidal shell that is twice as high as the spherical one is also twice as stiff.

**Figure 1:**
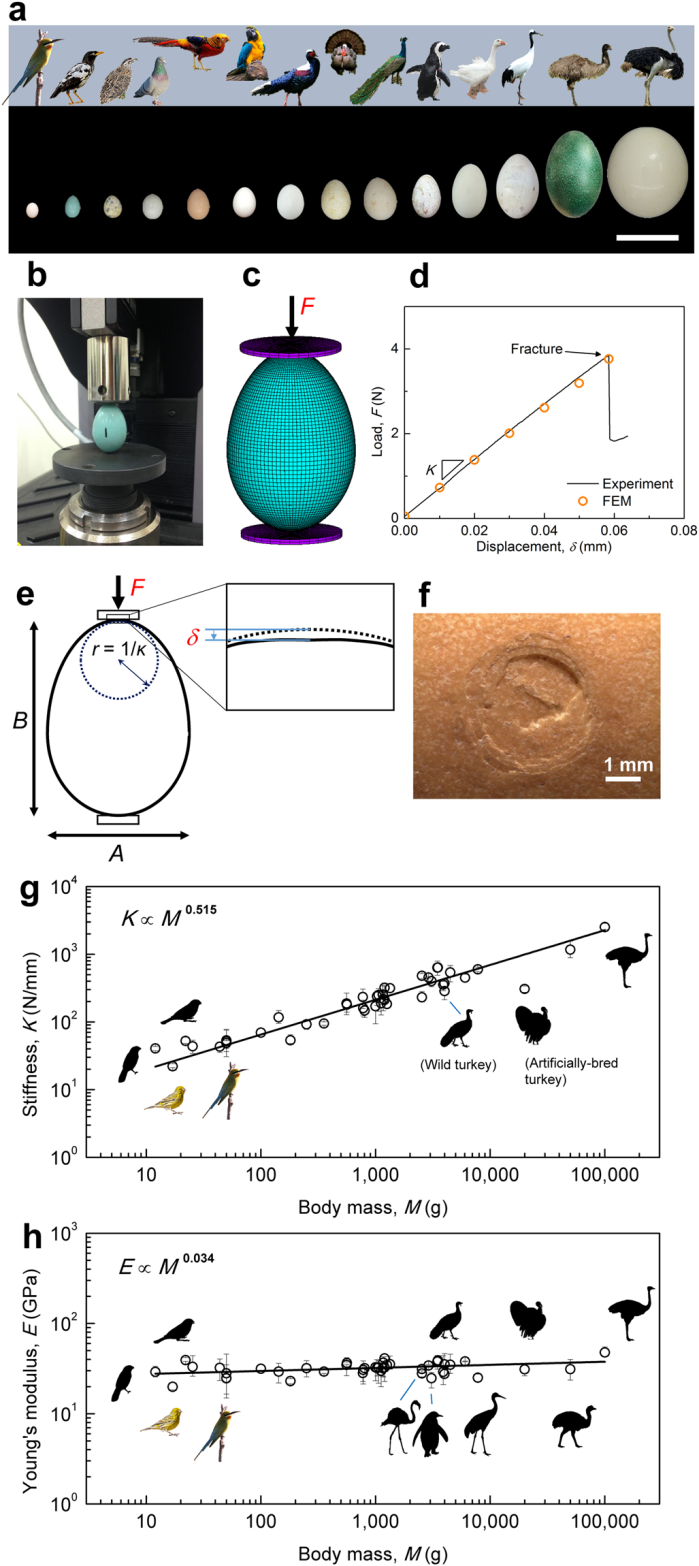
Astonishing variety of sizes and shapes of avian eggs. (**a**) Comparison of 14 representative eggs. What do they have in common? scale bar: 100 mm. Left to right: Blue-tailed bee-eater (*Merops philippinus*), Javan myna (*Acridotheres javanicus*), common quail (*Coturnix coturnix*), rock dove (*Columba livia*), golden pheasant (*Chrysolophus pictus*), Blue-and-yellow Macaw (*Ara ararauna*), Swinhoe’s pheasant (*Lophura swinhoii*), wild turkey (*Meleagris gallopavo*), Indian peafowl (*Pavo cristatus*), African penguin (*Spheniscus demersus*), domestic goose (*Anser sp*.), red-crowned crane (*Grus japonensis*), emu (*Dromaius novaehollandiae*), and ostrich (*Struthio camelus*). (**b**–**d**) Representative experimental setup showing a javan myna egg under compression test (**b**), its corresponding FEM model (**c**), and the load-displacement curve (**d)**. (**e**) Schematic of a deformed egg sample under compression test. (**f**) Representative image of a fractured egg of silver pheasant (*Lophura nycthemera*) after the compression test. The fracture almost always occurs at the top pole and exhibits a pattern known as “ring cracks”^37^, which are small compared to the egg length (≈50 mm). (**g**) Experimental stiffness along the long axis. (**h**) Young’s modulus *E*. The *E* of each egg sample is estimated by simulating the compression test by FEM. Our results reveal that *E* is quite constant (32 ± 5 GPa) for a wide range of body mass (12−100,000 g) and shell thickness (80−1,730 μm). The moduli of some species, however, are considerably larger or smaller than the average, e.g., the ostriches (48 GPa, *n* = 7) and African penguins (23 GPa, *n* = 4), which may be related to differences in the ultrastructure and composition of the shells (Supplementary Fig. S9; Supplementary Information). Error bars are the intraspecific maximum and minimum values. Bird images and silhouettes not to scale. See Supplementary Dataset 2 for details on the source of bird images. The egg images were taken by the authors.

Since the stiffness is a function of shell thickness, Young’s modulus, egg size and aspect ratio, this poses a fundamental challenge of quantifying eggshell stiffness in a way that allows meaningful intraspecific and interspecific comparisons. For example, the stiffness of an elephant bird egg is much larger than that of a hummingbird egg, but is it large enough to withstand the massive incubating bird? Here we ask whether there exist general “guidelines” that dictate the mechanical design of avian eggs. We propose a dimensionless number $C\equiv \frac{K}{W}\frac{{A}^{2}}{B}$, where *K* is the stiffness (N m^−1^) along the long axis, *A* and *B* the breadth and length of the egg, respectively, *W* the egg weight (N), and *A* ^2^/*B* a size/shape factor (see Table 1 for terminology and definitions). This approach has the important property of quantifying diverse eggs using a single metric with clear physical meaning—how stiff an eggshell is with respect to its egg size after removing the GIR^14^. Thus, eggs with dramatically different shapes, sizes and material properties can be compared (Fig. 1a).

**Table 1:** Terminology and nomenclature.

	Terms	Symbols	Definition	Determined by
Method 1: Freshly laid egg samples	The C number	*C*	$C\equiv \frac{K}{W}\frac{{A}^{2}}{B}$	Definition
	Shell stiffness	*K*	The initial slope of the experimental load-displacement curve	Experiment (compression test)
	Egg weight	*W*	The fresh egg weight	Experiment (digital scale)
	Egg breadth	*A*	The maximum lateral diameter of the egg	Experiment (vernier caliper)
	Egg length	*B*	The maximum length of the egg	Experiment (vernier caliper)
	Young’s modulus	*E*	The Young’s modulus with which the simulated load-displacement curve coincides with the experimental one is regarded as the Young’s modulus of that particular eggshell	Fitting experimental data by FEM
	Compressive fore	*F*	The load applied to the egg	Experiment (compression test)
	Displacement	*δ*	The deformation of the egg due to the compressive force	Experiment (compression test)
Method 2: Published egg images and data	The *C* number	*C*	$C\equiv \frac{K}{W}\frac{{A}^{2}}{B}$	Definition
	Shell stiffness	*K*	The initial slope of the simulated load-displacement curve	FEM
	Egg weight	*W*	The fresh egg weigtht	ref.^16^
	Egg breadth	*A*	The maximum lateral diameter of the egg	refs^11,16^
	Egg length	*B*	The maximum length of the egg	refs^11,16^
	Shell thickness	*t*	Shell thickness (without membrane)	refs^16^
	Young’s modulus	*E*	Elastic constant used in the FEM simulations	30 GPa (assumed)
	Compressive fore	*F*	The load applied to the egg	FEM
	Displacement	*δ*	The deformation of the egg due to the compressive force	FEM
	Aspect ratio	*B/A*	Relatively round eggs have small values, e.g. elf owl = 1.16; elongate ones have larger values, e.g. maleo = 1.71.	Definition
	Critical dimensionless number	*C*_cr_	${C}_{{\rm{cr}}}\equiv \frac{{K}_{{\rm{cr}}}}{W}\frac{{A}^{2}}{B}$	Definition
	Factor of safety	*F.S*.	*F.S*. ≡ *C*/*C*_cr_; *F.S*. is used to quantify the load-bearing capacity of an egg beyond the expected loads from incubating bird.	Definition
	Fracture force	*F*_f_	The load at which the egg fractures	Experiment (compression test)
	Buckling force	*F*_b_	The load at which the egg buckles	FEM
	Critical stiffness	*K*_cr_	The stiffness of a shell with thickness *t*_cr_	FEM
	Curvature	*κ*	Local curvature at the pole	2*B*/*A* ^2^ (ref.^14^)
	Mean curvature	*κ*_*M*_	Mean curvature of two principal curvatures	Definition
	Body mass	*M*	Body mass of adult bird (average of male and female)	refs^25,39^
	Radius of curvature	*r*	Radius of curvature at the pole, *r* = 1/*κ*	*A* ^2^/2*B*
	Fracture strength	*σ*_f_	The maximum stress in the shell when the egg fractures	FEM simulation using experimental fracture force
	Critical thickness	*t*_cr_	The thickness at which a given shell, subject to the weight of incubating bird, just begin to buckle (Supplementary Fig. S8b)	FEM
	Poisson’s ratio	*ν*	The ratio of lateral to longitudinal strain under the condition of uniform and uniaxial longitudinal stress	0.3 (assumed)

Here we study 463 bird species in 35 extant orders and one extinct order (Supplementary Dataset 1). We use two methods to determine shell stiffness *K*: (i) In Method 1, we experimentally compress freshly laid egg samples, and (ii) in Method 2, we create finite element models of eggs using published egg images and data, and run numerical simulations, resembling the compression tests. Once *K* is determined, the C number is determined by definition.

## Results

### C number based on freshly laid eggs (Method 1)

We experimentally compressed over 400 freshly laid intact egg samples from 40 bird species (11 orders, 16 families; Supplementary Dataset 1) using quasi-static compression tests (Fig. 1b, Supplementary Fig. S2), and obtained their stiffness *K*, defined as the initial slope of the load-displacement curve (Fig. 1d). We used this method for two reasons. First, this is a standard measurement used by the poultry industry so the measured stiffness is very repeatable, accurate, and with high resolution. There is no black box nor hidden parameters that might affect the results, once the apparatus is properly calibrated (Supplementary Information). Second, this measurement gives us the local stiffness of the shell near the contact area (approximately a few mm for a chicken egg) between the shell and plates. This stiffness indicates how much a shell may deform at the contacting regions due to an external force, be it the weight of incubating bird, the impact from other eggs in the nest, or the loading of a rigid plate in the experimental apparatus. The external force applied by the standard compression test mimics the selection pressures experienced by wild birds since their eggs constantly experience external forces.

To obtain the Young’s modulus, *E*, for each eggshell, we performed FEM simulations (Fig. 1c) using the same loading and boundary conditions as those used in the compression tests. The Young’s modulus at which the simulated load-displacement curve coincides with the experimental value is regarded as the Young’s modulus of that particular eggshell (Fig. 1d). Note that the determination of C number does not require the Young’s modulus, since $C\equiv \frac{K}{W}\frac{{A}^{2}}{B}$, where *K, W, A*, and *B* are all measured directly. The Young’s modulus is largely invariant, with an average value of *E* ≈ 32 ± 5 GPa, for the 40 species studied here (Fig. 1h; Table 2; Supplementary Dataset 1). This is an important result. Since most studies in the literature focused on domestic fowls^13^, the Young’s moduli of wild birds were previously unexplored.

**Table 2:** Mechanical qantities exhibiting allometric invariances, and their corresponding values and allometric exponents.

Mechanical quantity	Variable	Unit	Value	Best fit	S.E.*	*N*
Dimensionless number (Method 1)	*C*		15,200 ± 4,400	*M* ^−0.062^	±0.02	40^a^
Dimensionless number (Method 2)	*C*		15,200 ± 5,300	*M* ^−0.074^	±0.02	430^b^
Young’s modulus	*E*	GPa	32 ± 5	*M* ^0.034^	±0.01	40^a^
Normalized strength	*σ*_f_/*E*		0.73 ± 0.15%	*M* ^−0.006^	±0.02	40^a^
(Thickness)^2^/Egg weight	*t* ^2^/*W*	MPa^−1^	0.22 ± 0.07	*M* ^−0.093^	±0.02	40^c^
			0.28 ± 0.10	*M* ^−0.073^	±0.03	47^d^
			≈0.23	*M* ^−0.065^	—	3,434^e^
Fracture force/Egg weight	*F*_f_/*W*		55 ± 20	*M* ^−0.085^	±0.02	40^a^
Fracture force/(Thickness)^2^	*F*_f_/*t* ^2^	MPa	261 ± 72	*M* ^−0.001^	±0.02	40^a^

^a^Experimental results based on 400 freshly laid egg samples (Method 1). ^b^Numerical results based on published egg images and data, assuming *E* = 30 GPa. (Method 2). ^c^Present study, thickness does not include membranes. ^d^Ar *et al*.^9^, thickness includes dried membranes. ^e^Ar *et al*.^9^, based on the data in Handbuch der Oologie^16^; thickness was estimated from the eggshell mass and surface area. *t* ^2^/*W* = 0.23 (evaluated at *W* = 10 g); elephant birds *t* ^2^/*W* = 0.16 and *W* = 9,120 g; vervain hummingbirds *t* ^2^/*W* = 0.33 and *W* = 0.34 g. *Standard error of the exponent of the best fit. *N*: Number of species.

**Figure 2:**
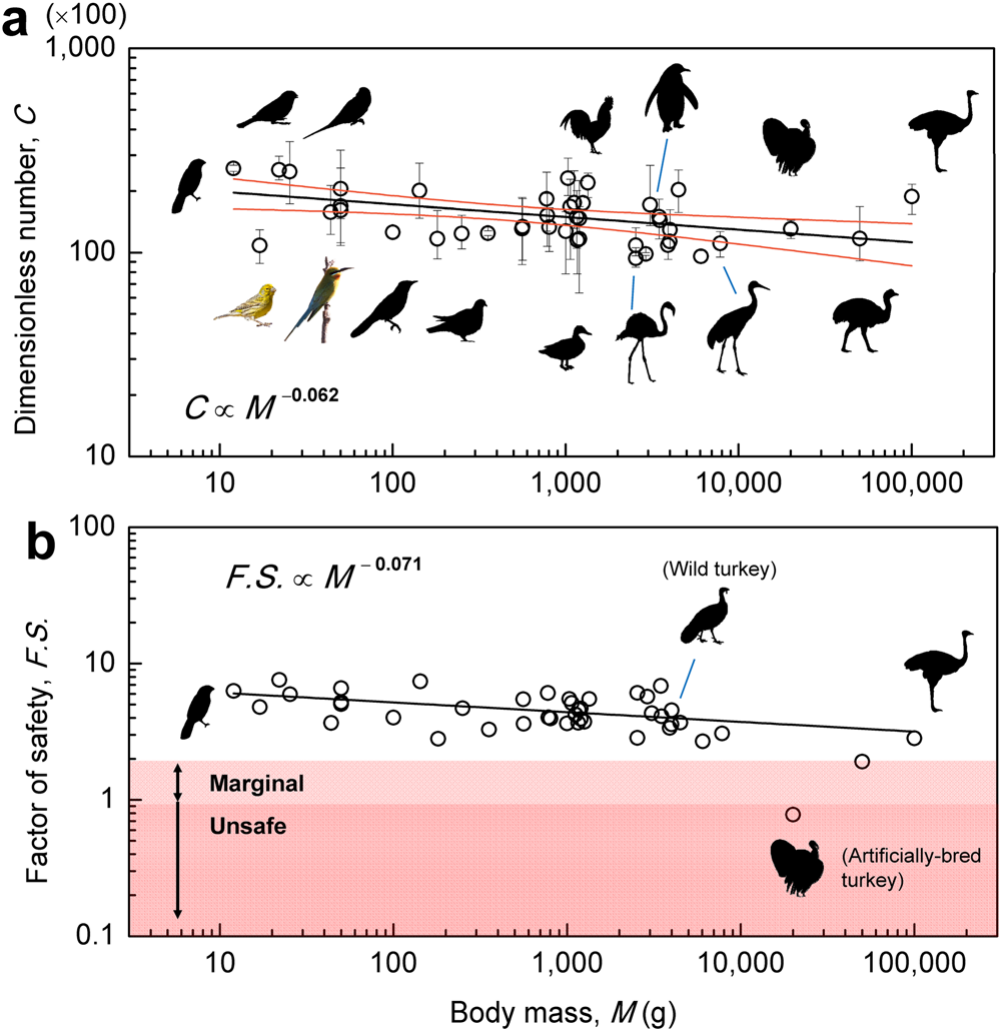
The C number and factor of safety based on freshly laid eggs (Method 1). (**a**) The experimental C versus body mass with one point per species. Symbols represent experimental measurements (error bars are the intraspecific maximum and minimum values), black line represents the best fit to the data, and red lines represent the 95% confidence intervals. (**b**) Factor of safety *F.S*. = *C*/*C*_cr_. Solid lines represent best fits to the data. Error bars are the intraspecific maximum and minimum values. See Supplementary Dataset 2 for details on the source of bird images.

Figure 2a shows that the C number remains nearly constant *C* = 15,200 ± 4,400 and *C* ∝ *M* ^−0.062^ across four orders of magnitude of body mass *M* from 12 to 100,000 g. This invariance is remarkable, considering that an ostrich’s egg (~1500 g) is nearly 830-fold heavier than a Scaly-breasted munia’s egg (~1.8 g). We map the experimental C numbers onto the phylogenetic tree, and observe that the C numbers are distributed between 10,000 and 20,000 (Supplementary Fig. S3). Substitution of basic scalings of *K*, *W*, *A*, *B* into C number yields *C* ≡ *K W* ^−1^ (*A* ^2^ */B*) ∝ *M* ^0.515^ *M* ^−0.756^ *M* ^0.257^ ∝ *M* ^−0.016^, indicating how the invariance of *C* is achieved.

We further quantify the safety margin of the eggs, subject to the force from the incubating bird, by defining a factor of safety *F.S*. (see Table 1 and more discussion below). The safety margin for contact incubation is categorized into three regions: safe (*F.S*. > 2), marginal (1 < *F.S*. < 2), and unsafe (*F.S*. < 1). All species in Fig. 2b have a *F.S*. greater than 2, except the artificially-bred turkey, which is included to serve as an interesting contrast to the wild turkey (see more discussion below).

### C number based on published egg images and data (Method 2)

**Figure 3:**
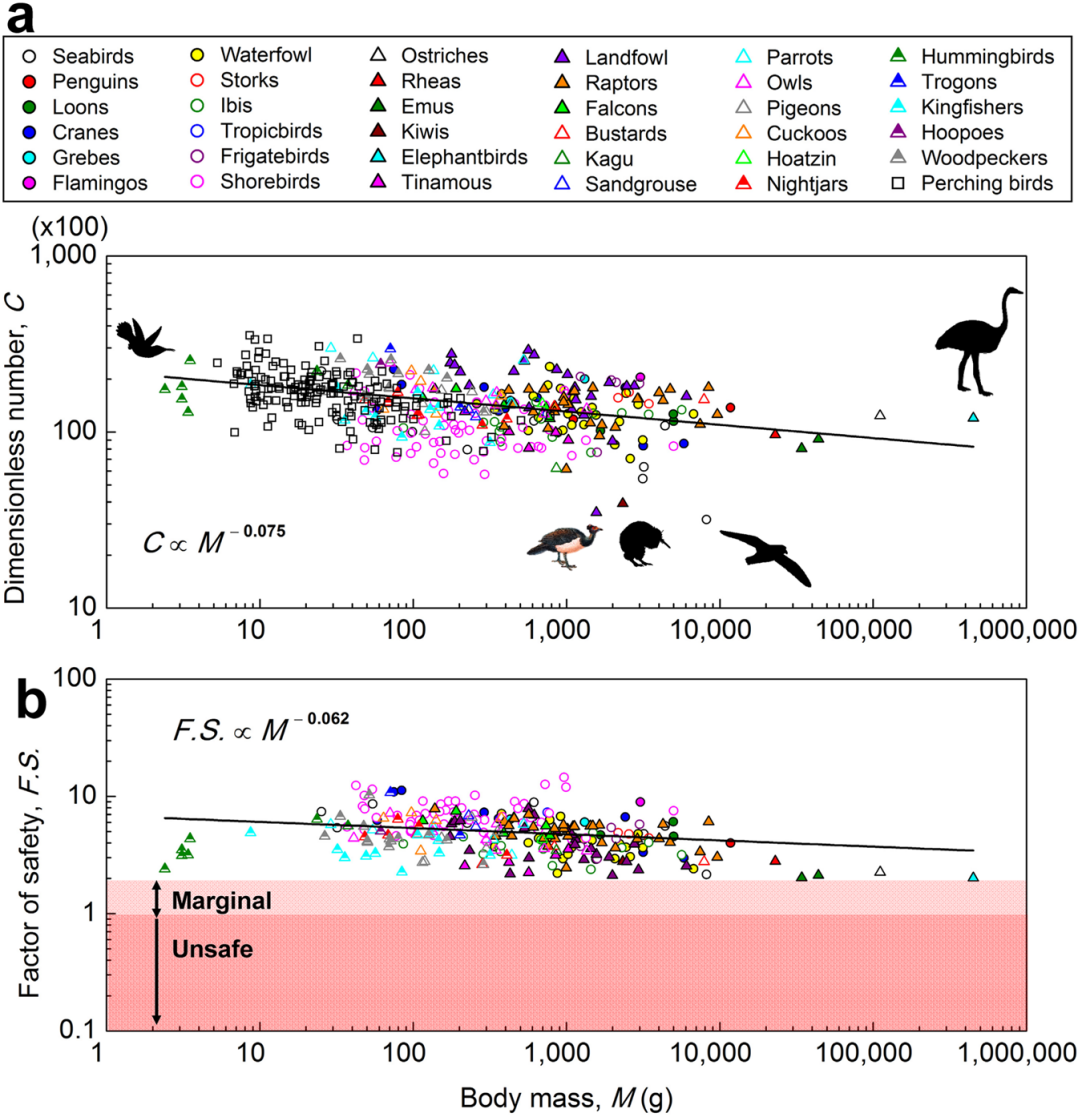
The C number and factor of safety based on published egg images and data (Method 2). (**a**) The C number of 430 species (36 orders, 104 families) predicted by the FEM simulations. The three species whose *C* depart substantially from the trend are maleos (*Macrocephalon maleo*), southern brown kiwi (*Apteryx australis*), and wandering albatross (*Diomedea exulans*). Their *F.S*., however, align well with other species. (**b**) Factor of safety, showing three regions: safe (*F.S*. > 2), marginal (1 < *F.S*. < 2), and unsafe (*F.S*. < 1). Each point represents one species, and each type of symbol represents one order. Assume *E* = 30 GPa for all species. The kiwis, megapodes and wandering albatross are not included in the calculation of the regression. See Supplementary Dataset 2 for details on the source of bird images.

We develop a new computational method (Methods and Supplementary Information) that enables us to study any egg provided that the egg profile, length, mass, shell thickness, and *E* are available. This approach greatly expands the number of species that one can study without the need to acquire freshly laid eggs, and proves useful to study the eggs of inaccessible and even extinct species. Here we extend our study to include 430 species (36 orders, 104 families; Supplementary Dataset 1) using published egg images^11^ and data^16^. We assumed a constant *E = *30 GPa for all simulations for simplicity. The stiffness *K* was obtained by performing compression simulations, resembling the experimental setup. The simulated *C* = 15,200 ± 5,300 and its scaling relation *C* ∝ *M* ^−0.075^ (Fig. 3a) are consistent with those obtained experimentally. Several species are observed to have very small C numbers, which will be discussed as special cases below, but their *F.S*. are all in the safe region (Fig. 3b).

### Evolution of body mass and C number

**Figure 4:**
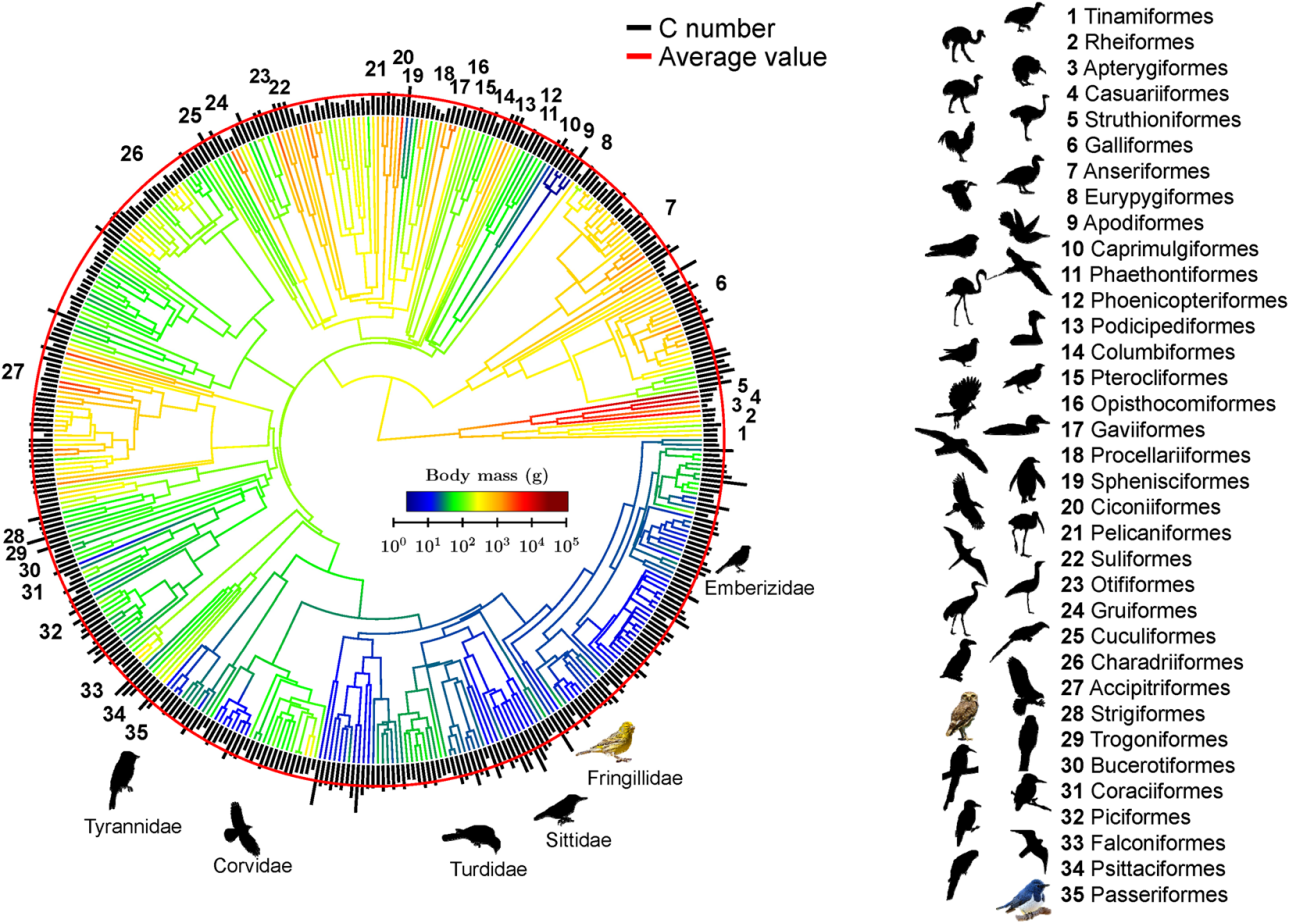
Evolution of body mass and C number. A phylogeny of 416 species, studied by Method 2, for which DNA sequencing data exist^18^. The C number for each species is represented by black line length (linear scale) at branch tips. The red circle indicates the average value (*C* = 15,000). The branch color indicates the body mass (log scale). Bird images for representative species in each order are shown (see Supplementary Dataset 2 for details). Bird images not to scale.

Body mass, or equivalently body size, is one of the most fundamental attributes of organisms, and influences many aspects of life^17^. As such, body mass is key to our understanding of egg evolution. Having quantified the egg stiffness using C number, we investigated the evolution of avian body mass and C number by assembling a recent comprehensive tree^18^ onto which we mapped body mass and C number of 416 species (35 orders) in our sample for which DNA sequencing data exist (Fig. 4). An investigation of the tree highlights three key observations.

First, body mass varies significantly across the tree, with small and large body masses occurring in parallel across different lineages. For example, extremely small body mass (<20 g) evolved independently in hummingbirds and in perching birds, whereas large body mass evolved in ratites and in some raptors. Second, the trend of body mass evolution is not singular. The body mass may monotonically increase such as ostriches, or decrease such as hummingbirds. It may also decrease and then increase such as cinereous vultures (*Aegypius monachus*), or increase and then decrease such as kiwis. Third, the body mass of the common ancestor of living birds, emerged around 113.2 million years ago^18^, is ~300 g (Fig. 4). Although fossil record of modern birds (Neornithes) from the Cretaceous Period is limited, the recently discovered fossils of Mesozoic birds, such as *Archaeornithura meemannae* ^19^ (Ornithuromorpha; ~77 g) and *Chongmingia zhengi* ^20^ (Ornithothoraces; ~290 g), corroborate our analysis and the view that small body size is common among birds throughout the Cretaceous Period^21^.

Despite the complicated evolutionary pattern of body mass, the C number, by contrast, remains largely invariant, suggesting that the “mechanical design” of avian eggs was well preserved throughout the process. We note that some clades, such as shorebirds (Charadriiformes), possess relatively small C numbers, which is due to their large eggs relative to body mass (≈15%). However, their *F.S*. are in the safe region (Fig. 3b).

Our analyses may shed light on the interesting evolution of kiwis and elephant birds. The elephant bird and kiwi are sister taxa^22^; they diverged ~50 million years ago and shared a common ancestor, which was probably flighted and capable of over-water dispersal. The body size of their ancestor was likely between those of the elephant bird and kiwi. We suggest that the gigantism of the elephant bird^22^ was accompanied with the gigantism of its egg to maintain a typical *W*/*M* ≈ 2%, whereas the kiwi egg did not shrink as much as the body mass did over evolutionary time, but instead the shell thickness was reduced to maintain a proper *F.S*.

### Mechanics of elastic shells

Why is C number nearly constant across a wide range of body mass? Consider an ellipsoidal shell loaded by the force *W* at its poles (local curvature *κ* = 2*B*/*A* ^*2*^). The shell is locally spherical and the indentation response is locally identical to the indentation of a spherical shell of radius $r=1/\kappa$ (ref.^14^). It follows that $C\equiv \frac{K}{W}\frac{{A}^{2}}{B}=\frac{K}{(\kappa /2)W}=\frac{K}{W}(2r)=\frac{2r}{\delta }=\frac{1}{\bar{\delta }}$, where $\bar{\delta }=\delta /2r$ is the normalized displacement. The C number of 15,000 indicates that $\bar{\delta }$ subjected to *W* falls within a certain range (~65 × 10^−6^). We substitute the vertical stiffness $K=[2E{t}^{2}/\sqrt{3(1-{\nu }^{2})}]\kappa$ of an ellipsoidal elastic shell^14^ into *C* and obtain $C\equiv \frac{K}{(\kappa /2)W}=\frac{1}{(\kappa /2)W}\frac{2E{t}^{2}}{\sqrt{3(1-{\nu }^{2})}}$ $\kappa =\frac{4}{\sqrt{3(1-{\nu }^{2})}}\frac{E{t}^{2}}{W}\propto \frac{E{t}^{2}}{W}$, where *ν* is the Poisson ratio. The factor *κ* in *C* eliminates the GIR, and allows for decoupling of contributions of shape/size and thickness/materials to the overall shell stiffness. Substituting for the measured allometric relations of *t*, *W*, *E*, and *M* from Fig. 1h and Supplementary Fig. S1, we arrive at *C* ∝ *t* ^2^ *W* ^−1^ *E* ∝ *M* ^0.732^ *M* ^−0.756^ *M* ^0.034^ ∝ *M* ^0.010^. The invariance of *C* is largely achieved by scaling *t* and *W* with respect to *M*, since *E* is found to be generally invariant.

### Fracture, buckling, and factor of safety

**Figure 5:**
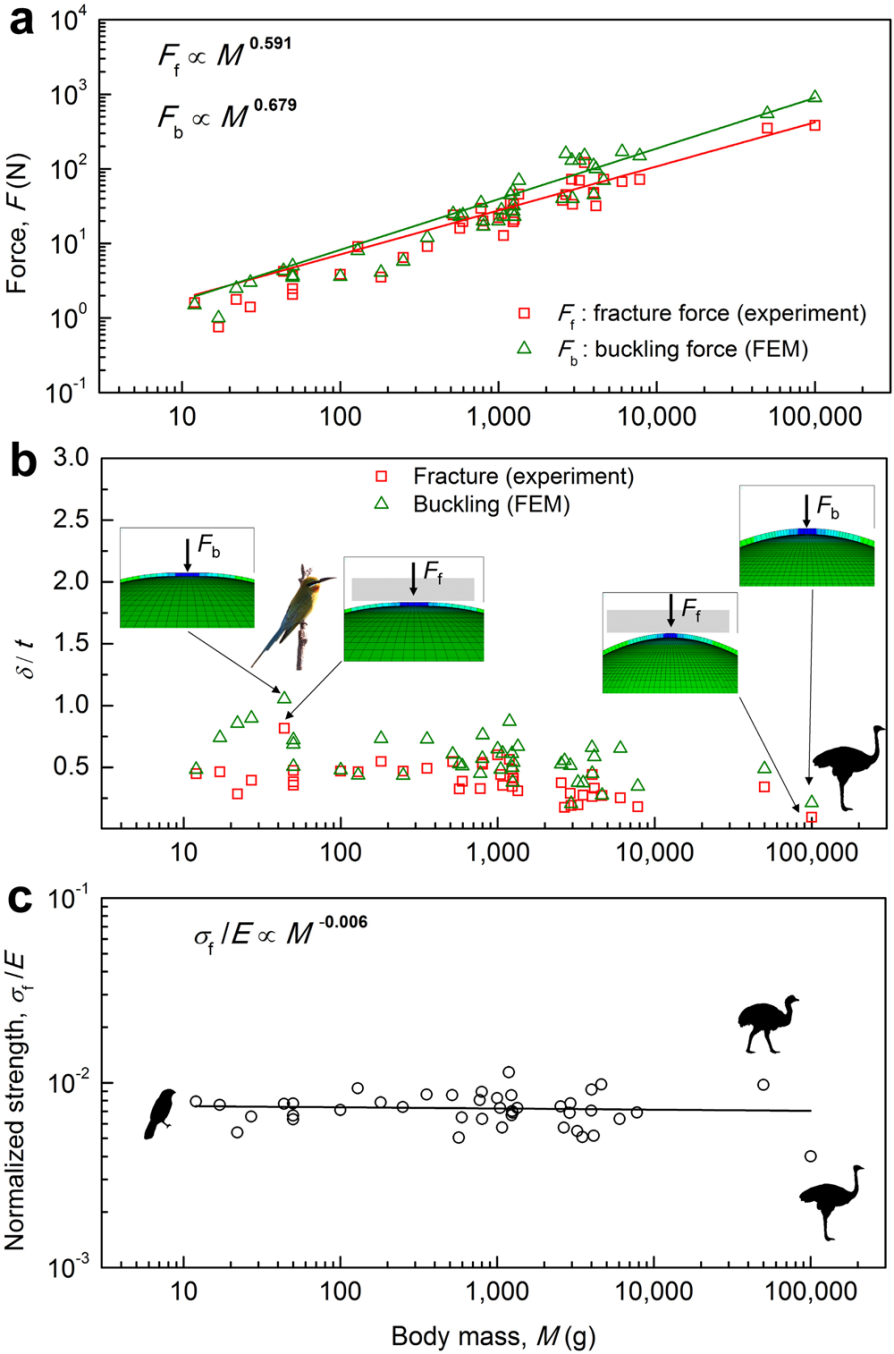
Failure characteristics of avian eggshells. (**a**) Experimental fracture force and simulated buckling force. (**b**) The displacement to shell thickness ratio *δ*/*t* at fracture (red squares) and buckling (green triangles). Much of the displacement comes from the bending deformation (flattening) near the contact zone. (**c**) Normalized fracture strength is approximately invariant: *σ*_f_/*E* = 0.73 ± 0.16%, or *σ*_f_ = *E*/137, see Fig. S7. A theoretical upper limit of *σ*_f_ is between *E*/2π and *E*/30 (ref.^38^). Fracture data are obtained from the compression test; buckling data are predicted by FEM simulations. See Supplementary Dataset 2 for details on the source of bird images.

Similar to engineering structures, the safety margin of the shell should be sufficiently high to account for environmental perturbations and incidental contact between eggs other than the static loading of incubating birds, but not too high to allow successful hatching and save of materials. Is there a lower limit for the thickness, and accordingly stiffness and C number, for a given body mass? We consider two failure modes: (i) fracture, and (ii) buckling. Figure 5a shows the experimental fracture force scales as *F*_f_ ∝ *M* ^0.591^ and simulated buckling force scales as *F*_b_ ∝ *M* ^0.679^. The buckling force is generally larger than the fracture force, indicating that the egg generally fractures before buckling, and buckling force may be used as an upper bound for the load an egg can withstand. Hence we use buckling as a criterion to estimate a lower limit for the shell thickness (critical thickness *t*_cr_), and in turn the critical stiffness *K*_cr_ and critical dimensionless number ${C}_{{\rm{cr}}}\equiv \frac{{K}_{{\rm{cr}}}}{W}\frac{{A}^{2}}{B}$ for a given body mass (Supplementary Fig. S4).

We define a factor of safety *F.S*. ≡ *C/C*_cr_ to quantify the load-bearing capacity of an egg beyond the expected load from the incubating bird. Factor of safety is often used in engineering with the intent to provide a safeguard to failure. The term usually refers to the ratio of the load that would cause failure of a structure to the load that is imposed upon it in service. The term may also be used to represent the ratio of the failure to service value of speed, deflection, temperature variation, or other stress-producing quantities.

The results shown in Figs 2b and 3b indicate that the *F.S*. decreases slightly with increasing body mass. This trend may be explained by the fact that smaller eggs (e.g. Scaly-breasted munia, *F*_f_ = 1.6 N) are more likely to be damaged by environmental disturbances or predator attacks than the larger eggs (e.g. ostrich, *F*_f_ = 383 N), and hence require extra safety margin.

Several other mechanical quantities are also found to exhibit allometric invariances irrespective of size and origin, and are summarized in Table 2.

### Case Studies

Several cases of special biological implications are elaborated in the following and are summarized in Table 3.

**Table 3:** Summary of case studies.

Species	*M* (g)	*E* (GPa)	*W* (g)	*t* (mm)	*C*	*F.S*.
Wild turkeys*	4,000	35	76	0.36	12,930	4.5
Artificially-bred turkeys*	20,000	31	84	0.39	13,060	0.8
Maleo (Megapodes)	1,564	30	222	0.38	3,489	3.2
Wandering albatross	8,190	30	455	0.58	3,186	2.16
Brown kiwi	2,330	30	434	0.50	3,900	2.8
Elephant birds	450,000	30	9,120	3.80	11,993	2.0
Vevain hummingbirds	2.4	30	0.34	0.033	17,400	2.4
Common cuckoos	112	30	3.3	0.10	19,400	3.4^a^/9.9^b^
White wagtails	21	30	3.0	0.08	17,353	3.7
Brown pelicans	3,147	30	112	0.47	13,728	3.8
Brown pelicans (DDE-affected)	3,147	30	112	0.31	6,527	1.8

*Experimental results. ^a^Incubated by cuckoos; ^b^Incubated by the host (white wagtails).

### Artificial selection

Artificial selection can produce breeds, all developed from the same wild species, that differ widely in appearance in just a few generations^23^. For example, the body mass of an artificially-bred turkey (*M* ≈ 20,000) is much larger than that of a wild turkey (*M* ≈ 4,000 g), which provides a unique contrast to test C number and *F.S*. We experimentally tested freshly laid eggs of both wild turkeys and artificially-bred turkeys, and found that, despite the great difference in *M*, their eggs are very similar in all aspects, including *C* ≈ 13,000 and *E* ≈ 33 GPa (Table 3), indicating that the shell mechanical characteristics remain unchanged through the artificial selection. The eggshell can no longer withstand the much larger body mass of the artificially-bred turkeys. The *F.S*. < 1 suggests that such a breed cannot survive without human assistance as the eggs must be artificially incubated.

### Extreme sizes

The recently extinct gigantic elephant bird and the vervain hummingbird are the largest known bird and the second-smallest living bird, respectively. Despite their extreme sizes, their *C* and *F.S*. are consistent with the overall trend. The *F.S*. of the five hummingbird species, ranging from 2.41 to 4.36, are slightly below the regression line due to the diminishing bending stiffness^24^, *B* = *Et* ^3^/12(1 − *ν* ^2^), at such a small thickness.

### Brood parasitism

The common cuckoo *Cuculus canorus* is a generalist obligate parasite, laying their eggs in nests of several passerine species, and has evolved numerous host-specific races, each having eggs with color and pattern mimicking the eggs of their respective host species^25,26^. Our results show that despite the fact that the cuckoo is four-fold heavier than one of its hosts, the white wagtail (*Motacilla alba*), their eggs are almost identical in appearance with cuckoo’s shell being slightly thicker. We obtain *C* = 19,400 and 17,353 for the cuckoo and wagtail, respectively. Interestingly, the *F.S*. of the cuckoo egg incubated by a wagtail (*F.S*. = 9.9) is significantly higher than that of the wagtail egg (*F.S*. = 3.7), supporting that parasitic cuckoo eggs are indeed stronger than the host’ eggs to withstand puncture rejection^27^.

### Loss of safety margin due environmental toxins

The C number may be used to quantify the detrimental impact of environmental toxins on eggshell stiffness and safety margin due to shell thinning^28^. Reproductive failure of brown pelicans (*Pelecanus occidentalis*) occurred in 1969 and a 35% decrease in shell thickness was associated with 71 p.p.m. of DDE in the egg^29^. Our simulation shows that such shell-thinning results in 53% drop in *C*, and accordingly, a significant drop of *F.S*. from 3.8 (safe) to 1.8 (marginal). The eggs were no longer strong enough for contact incubation.

### Exceptions

The C numbers of megapodes, kiwis, and wandering albatrosses are substantially smaller than the norm (Fig. 4a) mainly because their shells are thin (hence *K* is small) with respect to their egg weight *W*. Since $C\equiv \frac{K}{W}\frac{{A}^{2}}{B}$, a smaller *K* results in a smaller C number. These three species are exceptions of the constancy of C number. However, it is interesting to note that their *F.S*. are in the normal range (>2), strong enough for contact incubation, because their eggs are large relative to body mass in comparison to the ratios for other birds (Supplementary Dataset 1). One question is: The megapode does not practice contact incubation; why is its *F.S*. still in the normal range (*F.S*. = 3.2) and not smaller to save more materials? The answer remains unknown, but we hypothesize that this may be related to megapodes’ large clutch size (8−12 eggs). The large clutch size increases the possibility of incidental contact between eggs, so that the shell must be sufficiently thick to avoid damage. We note that all three species have the longest incubation period among birds (77 days, 74–84 days, and 60 days, for wondering albatrosses, the kiwis, and megapodes, respectively). The precise causality of the ecological adaptation of long incubation and small C number is unknown, and requires further studies. Nevertheless, the C number offers a single quantitative measure to identify species with special reproductive strategies.

## Discussion

The avian egg provides an excellent model system in which to explore the structural evolution of load-bearing biological structures. We define the C number, a measure of how stiff an egg is with respect to its weight after removing the shape effect, and find that it is generally invariant, suggesting that bird eggs are effectively working under a particular “design” irrespective of size or origin. This invariance is the result of two largely invariant mechanical quantities: *E* and *t* ^2^/*W*. Our conclusion is supported by the large dataset complied by Ar *et al*.^9^—they analyzed 3,434 species (over one-third of all living bird species) and showed that *t* ^2^/*W* is an allometric invariant (Table 2). From a biological perspective, as the body mass of a bird evolves to fit into a particular ecological niche, its egg weight and shell thickness evolve accordingly to maintain a constant *t* ^2^/*W* and a proper *W*/*M* ratio, which ensures safe contact incubation and saves precious shell materials. Similar allometric invariances are ubiquitous in nature. Examples include the urination time of large animals^30^, the Young’s modulus to density ratio in green woods^31^, and the height of a jump^32^. The existence of such invariances is mainly driven by the predominate force on earth —the gravity.

Considering the many requirements that the egg must fulfill, it is not surprising that the diversity of eggs is based on some elaboration of an “optimal design”—an optimal compromise between different selection pressures. Given the need to redesign the entire system whenever body mass changes, either through ontogeny or speciation, certain deviations from the general trend, and even exceptions, will occur. However, as body mass varies across many orders of magnitude, these design guidelines are followed with remarkable precision. Our findings presented here can be tested and provide a rich arena for future study on other amniote eggs^33^, structure-function relationship of eggshells^34^, avian reproductive strategies, and the evolution of amniote eggs and contact incubation^35^.

## Methods

### Egg Collection and Basic Measurements

Eggshell stiffness was measured in over 400 freshly laid intact eggs, belonging to 40 species from 15 families and 11 orders of birds, with egg mass ranging from 1.8 g (Scaly-breasted munia) to 1460 g (ostriches). Most egg samples were collected from the Taipei Zoo and some were acquired from captivity (Supplementary Dataset 1). Egg samples were photographed and the images were used to extract the eggshell profiles in SolidWorks (Dassault Systemes, Waltham, MA). Those profiles were later used in the finite element method (FEM) simulations package ANSYS. The basic egg properties were measured before compression tests. Egg mass (*W*) was measured by a digital scale; length (*B*) and breadth (*A*) were measured by a vernier caliper. After the compression test, three small fragments of each eggshell (one near the pointed pole, one near the blunt pole, the other near the equator) were prepared, and the shell thickness was measured per fragment, with and without shell membrane, using a digital tube micrometer (Mitutoyo 395–271, 0−25 mm range, 1 μm resolution with spherical/spherical anvils). We assume that the membrane has negligible contribution to the stiffness of whole egg, and does not include it when using the FEM simulations to obtain the Young’s modulus *E* of the shell.

All methods were carried out in accordance with relevant guidelines and regulations. All experimental protocols were approved by the Institutional Animal Care and Use Committee (IACUC) at National Taiwan University.

### Quasi-Static Compression Tests (Method 1)

We vertically compressed the freshly laid egg along its long axis, with its pointed end facing up, using an electromechanical universal test system (MTS Criterion Model 42) at room temperature. The egg was placed between two smooth steel plates. The bottom plate was fixed; the top plate was connected to a 250-N load cell. The loading was conducted at a constant compression speed of 1 μm s^−1^ until the egg fractured (Fig. 1 and Supplementary Fig. S2). The compressive load *F*, resulted from the compression by a prescribed displacement *δ*, was recorded using the load cell. Representative load-displacement curves are shown in Supplementary Fig. S2. The load-displacement curve was, in general, very linear until the egg fractured, at which a sudden drop in the load was observed and was often accompanied by a cracking sound. Examination of the tested sample shows that the fracture almost always occurred at the pointed pole, where concentric or radial cracks were formed (Fig. 1f) due to the high tensile stress generated on the inner surface of the shell during the test (Supplementary Fig. S7). The load at the first fracture was denoted as fracture force *F*_f_. We define the experimental stiffness *K* as the initial slope of the load-displacement curve. Although we compressed the egg sample until it fractured to measure the fracture force, the shell stiffness and C number can be measured using a small force without breaking the shell for nondestructive diagnosis.

### Numerical compression simulations (Method 2)

In Method 2, we analyzed egg stiffness of 430 species, covering 36 orders and 104 families, by numerical FEM simulations using published egg images^11^ and data^16^. To construct the model, we need the two-dimensional (2D) egg profile, egg length and mass, shell thickness, and Young’s modulus of the shell. The Young’s modulus of most avian species is absent in the literature; only a few common species have been studied^13^. In the present study, we found that the Young’s modulus is largely invariant, with an average value of *E* ≈ 32 ± 5 GPa, for the 40 species that we have tested (Table 2). For all simulations in Method 2, we assume a constant *E* = 30 GPa for simplicity. This approach greatly expands the number of species that one can study without the need to acquire freshly laid eggs, and proves useful to study the eggs of inaccessible and even extinct species. The stiffness *K* was obtained by compression simulations of the egg model with loading and boundary conditions resembling the experimental setup (Fig. 1c). To validate this approach, we randomly selected seven species and compared the egg characteristics with experimental results (Supplementary Table S1). Most parameters are, in general, in good agreement for the two methods. In particular, the deviation of the C number for 6 of the 7 species is within 25%. The only species whose *C* shows marked deviation is the ostrich, which is due to the difference in the Young’s modulus (30 and 48 GPa for Method 2 and Method 1, respectively). If *E* = 48 GPa is used, Method 2 predicts *C* = 19,700, which is very close to the average experimental value of 18,800 (<4.8%). This agreement is remarkable, considering the dramatically different nature of these two methods and substantial variation between species. This confirms that Method 2 is adequate for studying the mechanical design of the eggs of inaccessible and extinct species.

### Finite Element Method (FEM) Simulation

We used FEM for four purposes: (i) estimating the Young’s modulus, *E*, of a particular eggshell by fitting its experimental load-displacement curve (Fig. 1d); (ii) performing numerical compression simulations (Method 2); (iii) predicting the buckling force, *F*_b_, and factor of safety, *F.S*. (Fig. 5a,b, and Supplementary Fig. S8); and (iv) calculating the stress distribution and fracture strength, *σ*_f_ (Fig. 5c, and Supplementary Fig. S7). The FEM simulations were cross-validated by experiments and shell theory (Tables S1 and S2, Fig. S6).

### Failure due to Buckling

Although fracture force is a more direct measure of the eggshell strength and can be obtained from compression tests, it is more difficult to predict (micro-crack initiation, crack propagation, macroscopic catastrophic rupture), whereas existing shell theory^36^ and FEM simulations allow for more accurate predictions of the onset of buckling and buckling force, *F*_b_. The prediction of minimum stiffness, defined as the critical stiffness *K*_cr_, is based on the following scenario: When an eggshell is subjected to a force equal to the body weight, the maximum thickness that induces the eggshell to buckle is the critical thickness *t*_cr_ (Supplementary Fig. S8b) that determines *K*_cr_ (Supplementary Fig. S4a). In this sense, we applied a point force *M* at the pole and determined *t*_cr_ by adjusting the shell thickness (Supplementary Fig. S8). We then created a new eggshell model with *t* = *t*_cr_, and conducted the compression simulations, resembling the experimental condition, to obtain *K*_cr_. The critical dimensionless number *C*_cr_ and factor of safety *F.S*. are readily obtained for this eggshell.

### Data Availability

The datasets generated during and/or analysed during the current study are available from the corresponding author on reasonable request.

### Ethics

All methods were carried out in accordance with relevant guidelines and regulations. All experimental protocols were approved by the Institutional Animal Care and Use Committee (IACUC) at National Taiwan University.

## Electronic supplementary material

Supplementary Information

Dataset 2

Dataset 1
