# Supplementary material for: The avian egg exhibits general allometric invariances in mechanical design
Source: Sci Rep. 2017 Oct 27;7:14205. doi: 10.1038/s41598-017-14552-0 (PMC5660176; doi:10.1038/s41598-017-14552-0)
Supplement: Supplementary file 1 — Supplementary Information [file 41598_2017_14552_MOESM1_ESM.pdf]

Supplementary Information

The avian egg exhibits general allometric invariances in mechanical design

Jia-Yang Juang[1*], Pin-Yi Chen[1†], Da-Chang Yang[1†], Shang-Ping Wu[1], An Yen[1], and Hsin-I Hsieh[2]

[1]Department of Mechanical Engineering
National Taiwan University
Taipei 10617, Taiwan
[2]Taipei Zoo
Taipei 11656, Taiwan

[†]These authors contributed equally to this work.

*Correspondence and requests for materials should be addressed to J.-Y.J.
(jiayang@ntu.edu.tw)

Methods

Egg Collection and Basic Measurements. We mainly focus our experiments on the species belonging to Palaeognathae and Galloanserae, with additional species from other orders for comparison, because: (i) they vary dramatically in body mass from 100-100,000 g, (ii) they include the largest known extant and extinct species, (iii) they are early diverging precocial species laying eggs on the ground or in unsophisticated nests, and (iv) availability of freshly laid egg samples. Eggshell stiffness was measured in over 400 freshly laid eggs, belonging to 40 species from 15 families and 11 orders of birds, with egg mass ranging from 1.8 g (Scaly-breasted munia) to 1460 g (ostriches). Most egg samples were collected from the Taipei Zoo and some were acquired from captivity (Supplementary Dataset 1). Egg samples were photographed by iPhone 6s (12-megapixel camera), and the images were used to extract the eggshell profiles in SolidWorks (Dassault Systemes, Waltham, MA). Those profiles were later used in the finite element method (FEM) simulations package ANSYS. The basic egg properties were measured before compression tests. Egg mass (W) was measured by a digital scale. Length (B) and breadth (A) were measured by a vernier caliper. After the compression test, three small fragments of each eggshell (one near the pointed pole, one near the blunt pole, the other near the equator) were prepared, and the shell thickness was measured per fragment, with and without shell membrane, using a digital tube micrometer (Mitutoyo 395-271, 0–25 mm range, 1 μm resolution with spherical/spherical anvils). The shell thickness (without membrane) is generally uniform across the entire egg with a variation < ±10%, and the average of the three points was taken as shell thickness t. The relationships between W, t and M ($W \propto M^{0.756}$ and $t \propto M^{0.366}$) are consistent with the pioneering work by Rahn *et al.* ($W \propto M^{0.770}$ and $t \propto M^{0.353}$) refs.1,2. We assume that the membrane has negligible contribution to the stiffness

of whole egg, and does not include it when using the FEM simulations to obtain the Young's modulus E of the shell.

* All taxa were identified to species-level. The collected eggs of all different color morph lovebird, java sparrow, rock dove were analyzed separately. The eggs of the Gallus sp. were collected from different breeds, including Japanese bantam. Because the breeds of Gallus sp. are diverse in their body size and other life history traits, it is likely the eggs will show different characteristics, therefore we also discussed their egg features separately.

Quasi-Static Compression Tests (Method 1). We vertically compressed the freshly laid egg along its long axis, with its pointed end facing up, using an electromechanical universal test system (MTS Criterion Model 42) at room temperature. The egg was placed between two smooth steel plates. The bottom plate was fixed; the top plate was connected to a 250-N load cell. The loading was conducted at a constant compression speed of 1 μm s^{-1} until the egg ruptured (Fig. 1b, Supplementary Fig. S2). The load cell was replaced with a 2000-N one for ostrich and emu eggs, which required larger forces to rupture, and was replaced with a 5-N one for eggs lighter than 5 g for maximum resolution. The instrument elastic compliances (inverse stiffness) associated with the load cells were carefully calibrated as 1/11.40, 1/1385, and 1/5905 mm N^{-1} for the 5-N, 250-N, and 2000-N load cells, respectively, and their contribution to the displacement was removed. The compressive load F, resulted from the compression by a prescribed displacement δ, was recorded using the load cell. Representative load-displacement curves are shown in Supplementary Fig. S2. The load-displacement curve was, in general, very linear until the egg fractured, at which a sudden drop in the load was observed and was often accompanied by a cracking sound. Examination of the tested sample shows that

the fracture almost always occurred at the pointed pole, where concentric or radial cracks were formed (Fig. 1f) due to the high tensile stress generated on the inner surface of the shell during the test (Supplementary Fig. S7). Samples with pre-existing cracks showed distinct load-displacement curves with much smaller stiffness, and were not included in the analysis. The load at the first fracture was defined as the fracture force and denoted as F_f. The fracture force follows the allometric relation $F_f \propto M^{0.591}$ (Fig. 5a), and the displacement at fracture to thickness ratio $\delta/t = 0.40 \pm 0.14$ ($N = 40$) (Fig. 5b). A δ/t ratio smaller than one confirms that the deformation is in the linear regime since the nonlinear regime (buckling) occurs at ~ $\delta/t = 1$ (ref. [3]). Fracture failure is sensitive to pre-existing defects and micro-cracks[4]; by using the plate compression, we restrict the deformation within a very small volume, and obtain the largest attainable fracture strength σ_f.

We define the experimental stiffness K as the initial slope of the load-displacement curve. Compression tests with the egg loaded horizontally were also conducted for some selected species. Representative experimental setups for vertical and horizontal loadings for mallard (*Anas platyrhynchos*) are shown with their corresponding finite element models and dimensionless numbers in Supplementary Fig. S5. Egg samples were compressed in each direction, and the results were as follows: $F_f = 24 \pm 4$ N, $K_V = 197 \pm 34$ N mm^{-1}, $C_V = 97 \pm 20$, and $E = 32 \pm 5$ GPa for the vertical loading ($n = 56$); $F_f = 21 \pm 6$ N, $K_H = 133 \pm 39$ N mm^{-1}, $C_H = 114 \pm 38$, and $E = 33 \pm 4$ GPa for the horizontal loading ($n = 26$). The subscripts V and H denote the variables associated with compression directions. Note that E cannot be measured directly and is obtained by fitting the load-displacement curve by FEM (see below). Also, E obtained from both directions give consistent values, indicating that the elastic properties near the equator and the poles are the same.

Although we compressed the egg sample until it fractured to measure the fracture force, the shell stiffness and dimensionless number can be measured using a small force without breaking the shell for nondestructive diagnosis.

Numerical compression simulations (Method 2). Our numerical method enables us to study any egg provided that the egg profile, dimensions, mass, shell thickness, and E are available. This approach greatly expands the number of species that one can study without the need to acquire freshly laid eggs, and proves useful to study the eggs of inaccessible and even extinct species. Here, we extend our study to include 430 species (36 orders, 104 families) using published egg images[5] and data[6].

We digitally scanned egg images of each species from the Book of Eggs[5] into 600 dpi PDFs using a FUJI XEROX DocuPrint CM305 multifunction printer. Each egg image was imported into SolidWorks, and the egg profile was fitted manually and saved as an IGES file, which was then imported into ANSYS to create the eggshell mesh. The eggshell thickness, egg mass and length were obtained from Handbuch der Oologie[6] (= Handbook of Oology), and the body mass from CRC Handbook of Avian Body Masses[7] or from the allometric scaling relation $W \sim M^{0.770}$ (ref. 1), or from Handbook of the Birds of the World Alive[8]. We assumed a constant E =30 GPa for all species studied for simplicity. The stiffness K was obtained by the compression simulation resembling the experimental setup. Note that Method 2 gives a single set of parameters for each species without information on the variation. To validate this approach, we randomly selected seven species and compared the egg characteristics between Method 2 and the experimental results (Method 1) (Supplementary Table S1). Most parameters are, in general, in good agreement for the two methods. In particular, the deviation of the

dimensionless number C for 6 of the 7 species is within 25%. The only species whose C shows marked deviation is the ostrich, which is due to the difference in the Young's modulus (30 and 48 GPa for Method 2 and Method 1, respectively). If E = 48 GPa is used, Mehod 2 predicts C = 19,700, which is very close to the average experimental value 18,800 (< 4.8%). This agreement is remarkable, considering the dramatic different nature of these two methods and substantial variation between species. This confirms that Method 2 is adequate for studying the mechanical design of the eggs of inaccessible and extinct species.

Finite Element Method (FEM) Simulation. We used FEM for four purposes: (i) estimating the Young's modulus, E, of a particular eggshell by fitting its experimental load-displacement curve (Fig. 1d); (ii) performing numerical compression simulations (Method 2); (iii) predicting the buckling force, F_b, and factor of safety, $F.S.$ (Fig. 5a, b, and Supplementary Fig. S8); and (iv) calculating the stress distribution and fracture strength, σ_f (Fig. 5c, and Supplementary Fig. S7). We simulate the quasi-static compression process of the egg under two different loading conditions: (i) two parallel plates, and (ii) one bottom plate and top point force, by using the finite element package ANSYS. The former is used to simulate the experimental condition, and the latter is used to predict the buckling failure. The load-displacement curves of both conditions are almost identical at small loads (linear regime), but deviate from each other at larger loads as the response becomes nonlinear. The eggshell model is created using the measured eggshell profile and the average thickness without the membrane. The three-dimensional (3D) eggshell and plates are meshed with 4-node structural shell element (SHELL181) and 20-node structural solid element (SOLID186), respectively. The contact interface between the eggshell and plate is modeled by a 3D node-to-surface contact pair

(CONTA175 and TARGE170), and is assumed frictionless. The element size is sufficiently small so that the numerical results are insensitive to further refinement of the mesh. The bottom of the bottom plate is fixed, and the top plate moves only in the vertical direction (Fig.1c). Both eggshell and plate are assumed linearly elastic, homogeneous and isotropic. The plate is made of stainless steel with Young's modulus E = 200 GPa and Poisson's ratio ν = 0.3. For the eggshell, the Young's modulus with which the simulated load-displacement curve coincides with the experimental one is regarded as the Young's modulus of that particular eggshell. For loading condition one, the load-displacement curve is obtained by prescribed-displacement static simulations in which the top plate compresses the eggshell at prescribed displacements up to the fracture point, and the applied load required for static equilibrium is calculated. Geometric nonlinearity, such as large rotation and large strain, is included. The egg content, i.e. yolk and albumen, is not included in the model since it has a negligible effect on the shell strength and stiffness[9].

We compare the stiffness obtained by FEM and shell theory, $K = \frac{4Et^2}{\sqrt{3\left(1-\nu^2\right)}}\frac{b}{a^2}$, where a and b are respectively equatorial circular radius and polar radius, using five different half shells. The half shells are fixed at the bottom and loaded by a point force F at the poles. Our results show that both methods yield consistent values (Supplementary Table S2). For the ideal hemispherical and hemiellipsoidal shells, the deviation between FEM and theoretical values are small (< 2%), indicating that our FEM models are accurate. Note that the theoretical expression was derived assuming thin spherical shell without nonlinear effect, and our numerical comparison showed that it overestimates the stiffness by 10% if the thickness to radius ratio is over 2% (data not shown). Certain inaccuracy may also be introduced if we apply

the expression for eggshells with non-ellipsoidal profiles. On the other hand, the FEM simulation is applicable to relatively thick and non-ellipsoidal shells and includes nonlinear effects.

The Young's modulus obtained by FEM is in good agreement with that predicted by shell theory $E = \frac{K\sqrt{3\left(1-\nu^2\right)}}{4t^2}\frac{A^2}{B}$, where K is the experimental stiffness, $A = 2a$, and $B = 2b$ (Supplementary Fig. S6b). The FEM values are slightly larger than the theoretical values due to the same reason described in the previous paragraph—the theory overestimates the stiffness and hence underestimates the Young's modulus for relatively thick shells.

Failure due to Buckling. Although fracture force is a more direct measure of the eggshell strength and can be obtained from compression tests, it is more difficult to predict (micro-crack initiation, crack propagation, macroscopic catastrophic rupture), whereas existing shell theory[3] and FEM simulations allow for more accurate predictions of the onset of buckling and buckling force.

The prediction of minimum stiffness, defined as the critical stiffness K_{cr}, is based on the following scenario: When an eggshell is subjected to a force equivalent to the body weight, the maximum thickness that induces the eggshell to buckle is the critical thickness t_{cr} that determines K_{cr} (Supplementary Fig. S4a). In this sense, we apply a point force M at the pole and determine t_{cr} by adjusting the shell thickness (Supplementary Fig. S8). We then create a new eggshell model with $t = t_{cr}$, and conduct the compression simulations, resembling the experimental condition, to obtain K_{cr}. The critical dimensionless number C_{cr} and factor of safety $F.S.$ are readily obtained for this eggshell.

Effect of Compression Direction (Vertical vs Horizontal). Alternatively, the compression test may be conducted with the egg sample positioned horizontally, with its equator in contact with the two rigid plates (Supplementary Fig. S5). The loading process is then approximated as indentation of a nonaxisymmetric convex shell at its equator, where the two principal curvatures $\kappa_1 = 1/b$ and $\kappa_2 = 1/a$ are in general not equal. Thus, replacing $\kappa = 1/r$ by the mean curvature κ_M (ref. 10), we obtain $C_H \equiv \frac{K}{(\kappa_M/2)W} = \frac{K}{W}(2r_M) = \frac{2r_M}{\delta} = \frac{1}{\bar{\delta}}$. In this case, the indentation is locally identical to the indentation of a spherical shell of radius $r_M = 1/\kappa_M = 2/(\kappa_1 + \kappa_2)$. Hence, $C_H \equiv \frac{K}{(\kappa_M/2)W} = \frac{K}{W}\left(\frac{2AB}{A+B}\right)$, where the shape/size factor becomes $(2AB)/(A+B)$. Similarly, for the horizontal loading at the equator, we substitute $K_H = \left[2Et^2/\sqrt{3(1-\nu^2)}\right]\kappa_M$ into C_H, and obtain $C_H \equiv \frac{K_H}{(\kappa_M/2)W} = \frac{1}{(\kappa_M/2)W}\frac{2Et^2}{\sqrt{3(1-\nu^2)}}\kappa_M = \frac{4}{\sqrt{3(1-\nu^2)}}\frac{Et^2}{W}$. The dimensionless number gives the identical result, $C \propto Et^2/W$, independent of the compression direction since the geometry-induced rigidity is removed. Indeed, we may conduct the compression test in either direction, and obtain the same dimensionless number (Supplementary Fig. S5e). Also, the Young's modulus can be directly estimated from the experimental stiffness using $K_V = \left[4Et^2/\sqrt{3(1-\nu^2)}\right](B/A^2)$, but since the actual eggshell profile is not ellipsoidal, certain error might be expected. Nevertheless, experiments, shell theory, and FEM give consistent results (Supplementary Fig. S6).

SEM images of eggshell ultrastructure, and factors affecting the mechanical properties

The eggshell is a highly ordered multilayer porous material largely made of calcite crystals (a polymorph of calcium carbonate, $CaCO_3$), embedded in an organic matrix, preferentially oriented with their *c*-axis perpendicular to the shell surface[11], and is mainly composed of four layers (from inner to outer surface): (*i*) the shell membrane, (*ii*) the inner mammillary cone layer adhered to the shell membrane, (*ii*) the columnar palisade layer, also known as spongy layer and squamatic ultrastructure[12], that makes up most of the shell material, and (*iv*) the thin, surface cuticle[13,14]. Examples of cross-sectional scanning electron micrographs (SEM) of the eggshells are shown in Supplementary Fig. S9. Some species, such as pigeon and budgerigar, were found to possess very thin or no cuticle[15]. The eggshells of most avian species have simple, straight pore canals that run through the shell thickness, enabling gas exchange[13]. Some species such as ratites possess pores branching from the origins near the shell membrane into a more complex network[16]. The palisade layer is also found to contain numerous spherical vesicles (voids) with various diameters (e.g. ~450 nm for chicken[14] and 1−2 μm for budgerigar[15]). The vesicles are not just air bubbles but filled with organic material connected by a continuous network of organic fibrils[17]. Although the shell is essentially anisotropic and nonhomogeneous, the exact modeling of its ultrastructure and materials is complicated; here, we simplify our FEM simulations by assuming an isotropic and homogeneous material, represented by the Young's modulus.

The mechanical properties of the shell are largely dependent on the relative amount of mineral and organic materials, size and number of vesicles, and the ultrastructure defined as the size, shape and crystallographic orientation of the calcite crystals[18]. Our SEM images reveal that the peafowl shell shows well-crystallized calcite crystals within the palisade layer. Aside

from minute vesicles, occasional dislocations, and pore canals, they are relatively defect-free, whereas the bee-eater shell is not as well-structured and contains larger and more densely distributed vesicles (Supplementary Fig. S9). Despite their distinct features, the Young's moduli of the peafowl shell (35 ± 5 GPa, n = 24) and bee-eater shell (32 ± 6 GPa, n = 9) only deviate from each other by ~10%, exhibiting similar macroscopic elastic properties.

Table S1. Comparison between Method 1 (experiments) and Method 2 (FEM simulations)

Species	Method	*W* (g)	*B* (mm)	*A* (mm)	*t* (mm)	*M* (g)	*K* (N mm^{-1})	F_f (N)	*E* (GPa)	*C*	*Ccr*	*F.S.*
Mallard	Exp.	59.70	61.14	42.12	0.28	1,200	192.07	23.26	31.8	9,803	2915	3.43
(*Anas platyrhynchos*)	FEM	54.0	56.8	41.2	0.31	1,082	198	–	–	10,940	2966	3.69
Wood duck	Exp.	44.03	52.26	39.33	0.25	570.0	194.15	23.27	36.5	13,121	2,372	5.58
(*Aix sponsa*)	FEM	44.0	51.2	38.8	0.32	658	181	–	–	12,081	2,752	4.39
Common emu	Exp.	631.6	132.78	90.27	1.03	50,000	1120.4	344.6	30.3	10,812	5,883	1.85
(*Dromaius novaehollandiae*)	FEM	610	136	89.0	0.94	34,200	842	–	–	8,044	3,982	2.02
Common ostrich	Exp.	1,466	147.7	126.9	1.73	100,000	2,516	396.7	47.8	18,797	6,538	2.83
(*Struthio camelus*)	FEM	1,600	159	131	1.92	111,000	1,828	–	–	12,369	5,482	2.26
Swinhoe's pheasant	Exp.	41.1	51.9	39.0	0.30	1,047	230	25.8	29.5	15,920	2,984	5.28
(*Lophura swinhoii*)	FEM	43.0	52.2	38.6	0.34	1,100	254	–	–	16,893	3,054	5.53
Wild turkey	Exp.	75.6	64.7	46.9	0.36	4,000	285	42.8	35.2	12,931	3,125	4.55
(*Meleagris gallopavo*)	FEM	71.5	62.0	45.8	0.39	6,050	337	–	–	15,960	6,259	2.55
Rock Dove	Exp.	18.4	38.7	30.2	0.18	250	92.07	7.6	31.9	12,379	2,479	4.70
(*Columba livia*)	FEM	17.8	39.1	29.1	0.18	355	110.7	–	–	13,465	2,688	5.01

Table S2. Comparison between FEM simulations and shell theory. *a* and *b* are equatorial circular radius and polar radius, respectively.

	Hemiellipsoidal	Hemispherical	Mallard	Bee-eater	Ostrich
	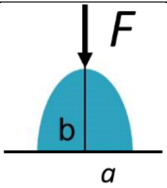<br>a = 25 mm<br>b = 50 mm<br>t = 0.1 mm	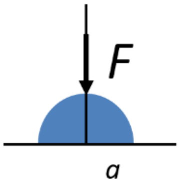<br>a = 25 mm<br>t = 0.1 mm	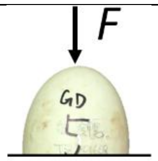<br>a = 21.7 mm<br>b = 31.3 mm<br>t = 0.288 mm	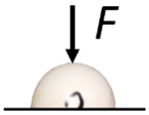<br>a = 10.3 mm<br>b = 11.4 mm<br>t=0.107 mm	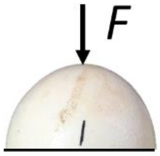<br>a = 63.9 mm<br>b = 73.2 mm<br>t = 1.7 mm
Shell theory	58.1 N mm^{-1}	29.1 N mm^{-1}	442.5 N mm^{-1}	101 N mm^{-1}	5363 N mm^{-1}
FEM	57.9 N mm^{-1}	29.7 N mm^{-1}	437.8 N mm^{-1}	98.4 N mm^{-1}	5363 N mm^{-1}
deviation	-0.3%	2.1%	-1.06%	-2.85%	-0.54%

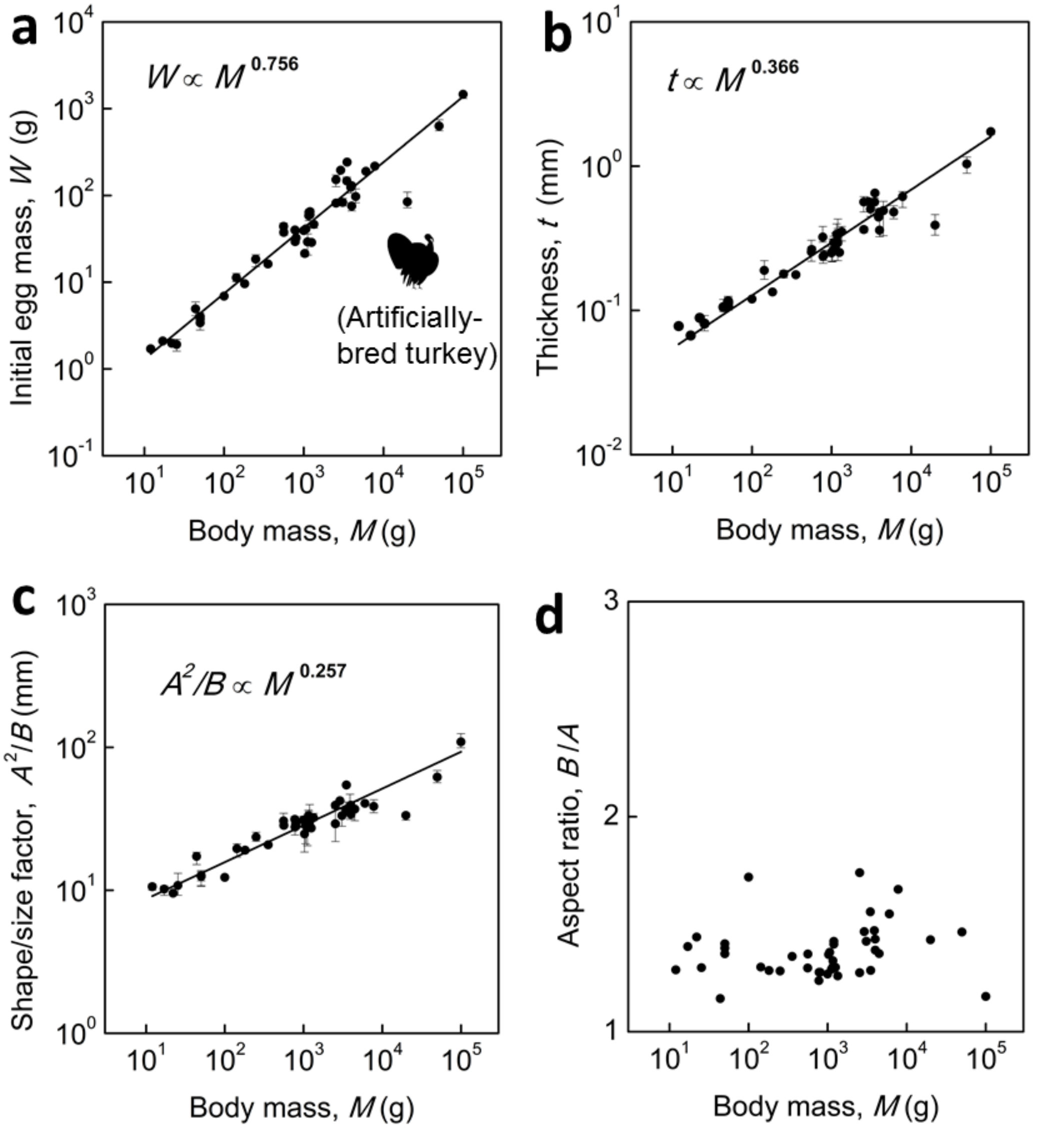

Figure S1. Allometric scaling relationships of basic egg properties. a, Egg mass *W*. Artificially-bred turkeys, e.g. Broad Breasted Whites[19], were bred from wild turkey specifically to produce maximum meat. They are often several times heavier than the wild turkey, and have a *W*/*M* ratio below the regression line. **b,** Shell thickness *t*. The allometric scaling relations of *W* and *t* versus *M* are consistent with the pioneering work by Rahn *et al*. [1,2]. **c,** Shape/size factor A^2/B. **d,** Aspect ratio *B*/*A*. No apparent relation exists, suggesting that egg shape may evolve according to factors besides load-bearing, such as 'packaging', rolling prevention[20,21], contamination-related effect[22], and even the flight ability of birds[23]. Solid lines represent best fits to the data. Error bars are the intraspecific maximum and minimum values.

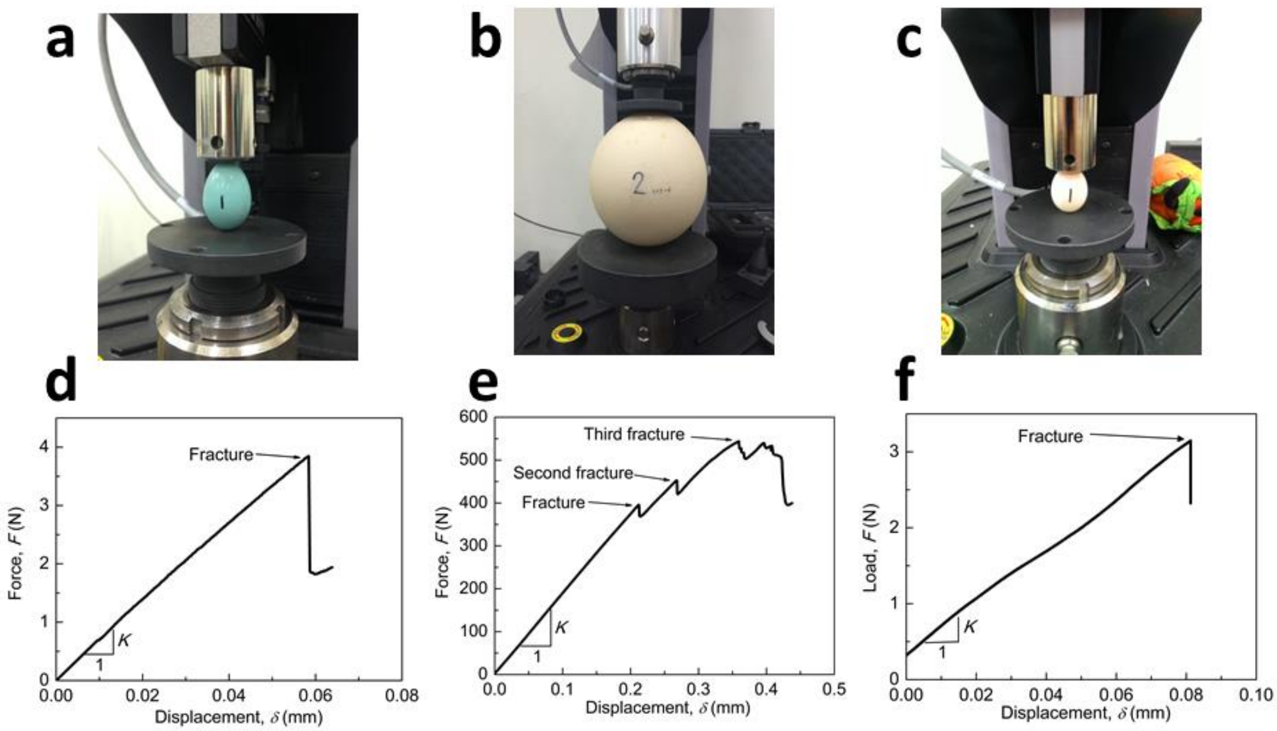

Figure S2. Quasi-static compression tests of freshly laid eggs. a-c, Experimental setups for javan myna (*Acridotheres javanicus*) (**a**), ostrich (**b**) and blue-tailed bee-eater (*Merops philippinus*) (**c**), and their corresponding load-displacement curves in **d-f**.

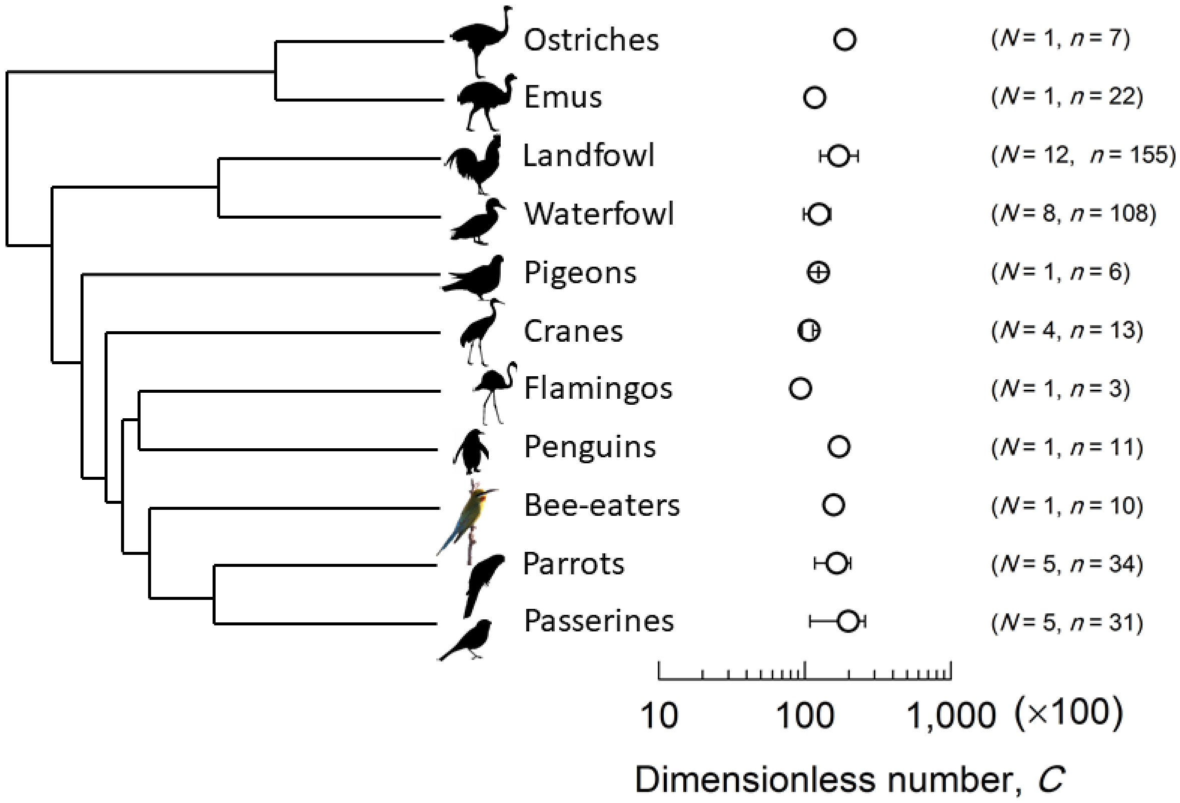

Figure S3. Phylogenetic tree[24] and the experimental C numbers. The results were obtained by experimentally compressing 400 freshly laid egg samples, which belong to 11 orders, 15 families, and 40 species. Horizontal bars are the interspecific maximum and minimum values in the same order; N: number of species, n: number of samples. See Supplementary Dataset 2 for details on the source of bird images.

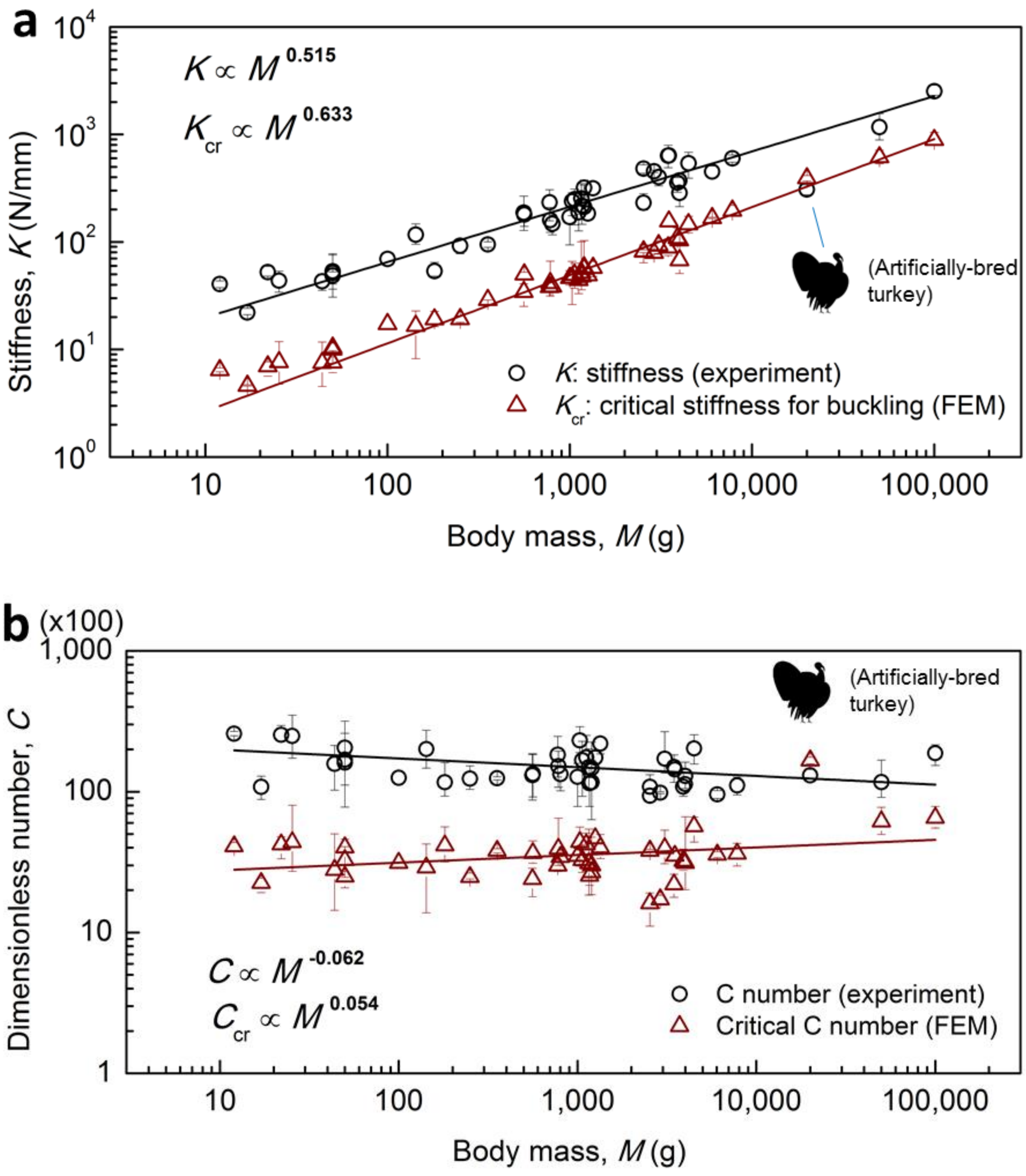

Figure S4. Critical stiffness and critical *C* number. a, Stiffness *K* and critical stiffness K_{cr}. **b,** The dimensionless number and its lower bound predicted based on buckling.

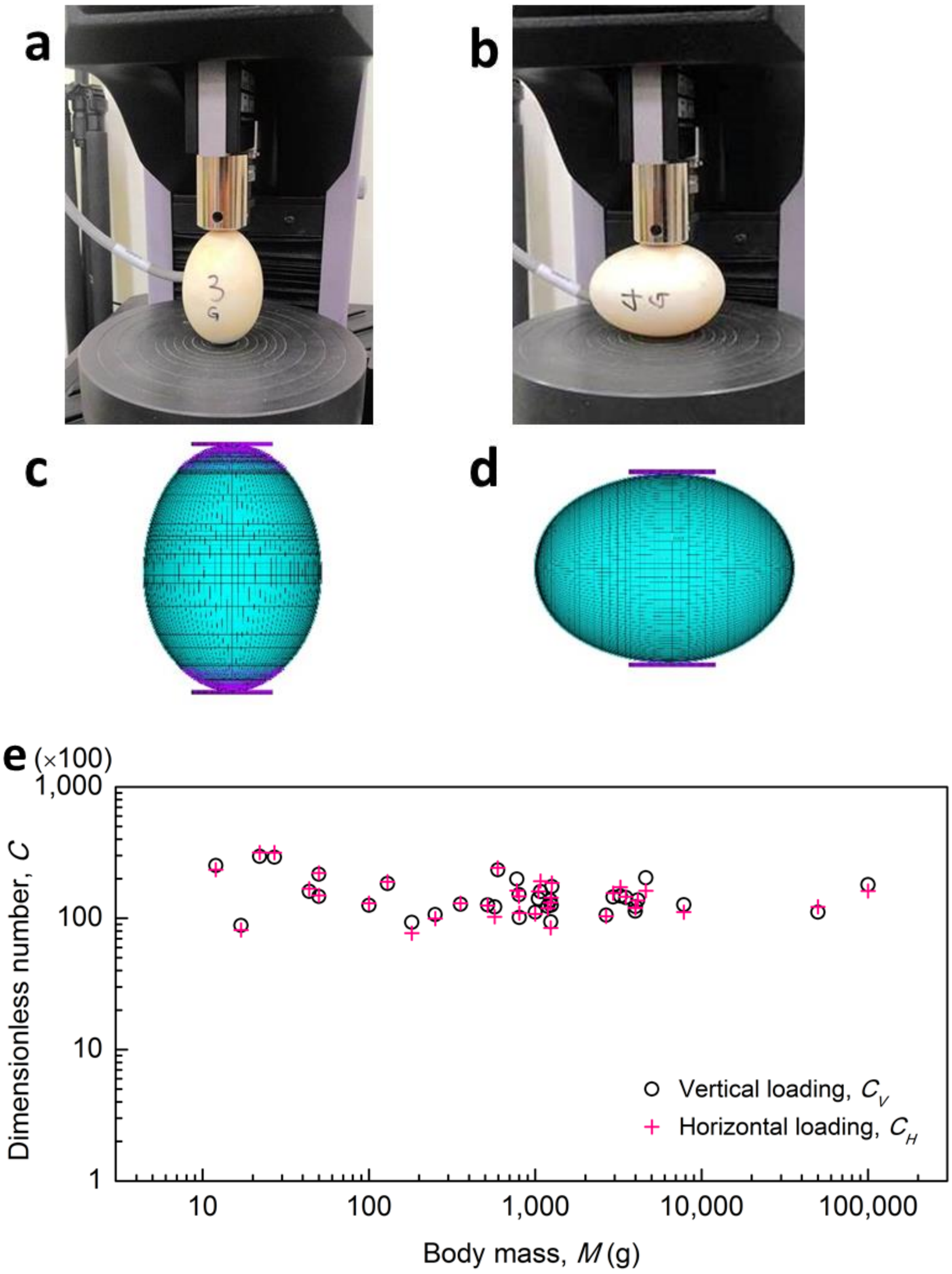

Figure S5. Effect of compression direction. a, b, Experimental setups for vertical loading (**a**) and horizontal loading (**b**), and their corresponding FEM models in **c, d**. **e,** Comparison of the dimensionless numbers obtained from vertical loading $C_v \equiv (K_v/W)(A^2/B)$ and horizontal loading $C_H \equiv (K_H/W)[2AB/(A+B)]$.

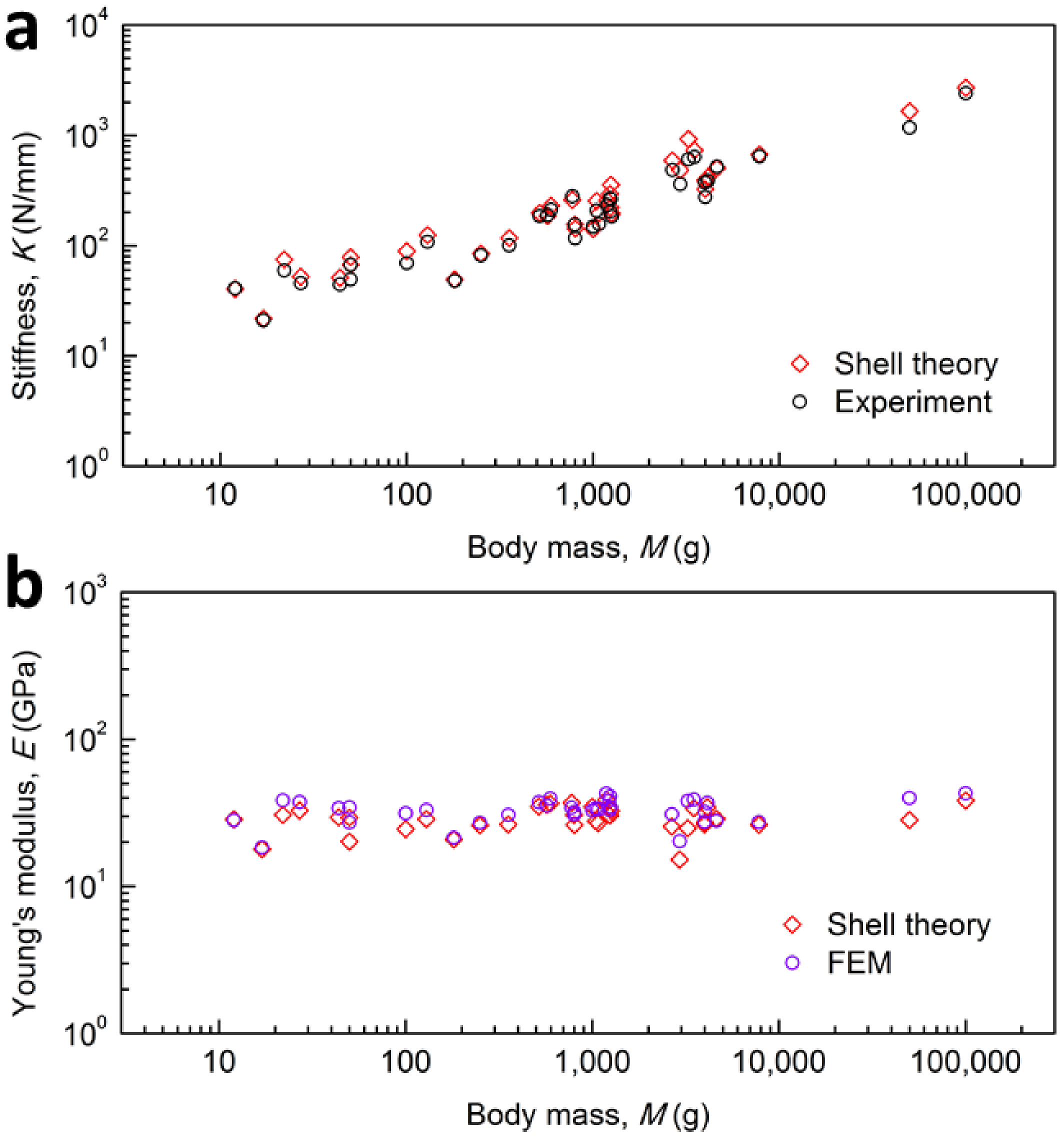

Figure S6. Cross validation between FEM simulations, shell theory, and experiments. a, Comparison of stiffness obtained by experiments and shell theory, $K = \left[4Et^2 / \sqrt{3(1-\nu^2)}\right] B/A^2$, in which E is estimated by fitting the experimental load-displacement curves using FEM. **b,** Comparison of E obtained by FEM and the same theory but expressed as $E = K\left[\sqrt{3(1-\nu^2)} / 4t^2\right] A^2/B$, in which K is from the experiments.

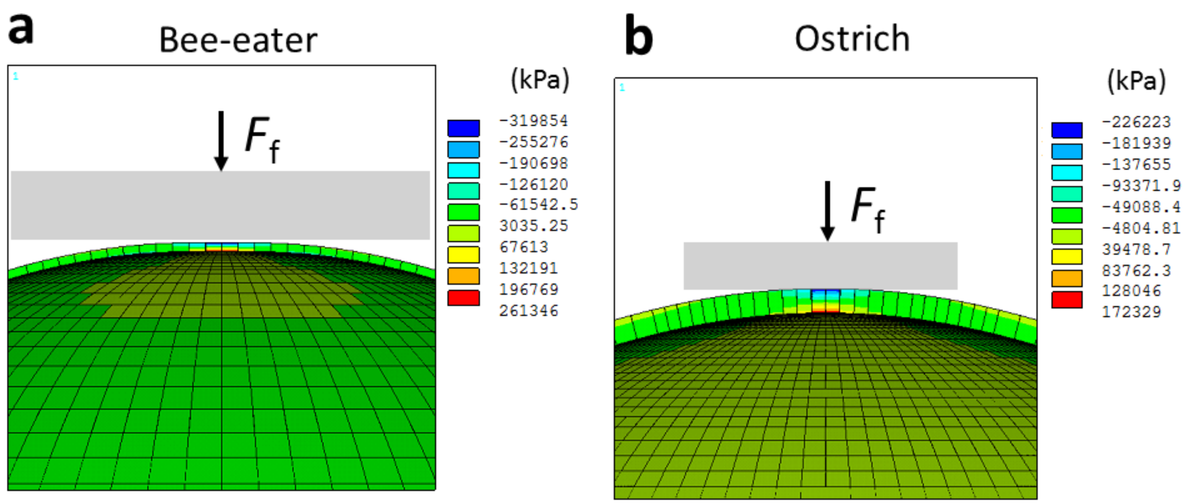

Figure S7. Examples of in-plane normal stress distribution at fracture. a, Blue-tailed bee-eater. **b,** Ostrich. σ_f is the in-plane tensile stress located at the inner surface of the top pole, where the crack initiates, consistent with our experimental observations and ref. [25]. The stress on the inner surface largely arises from the bending moment due to flattening[26], and also has contributions from the Hertz contact[27]. When the pole is flattened to conform to the rigid plate, the biaxial bending moments and the corresponding biaxial tensile stress on the inner surface are respectively $M = Et^3/[12(1 - \nu)\ r]$ and $\sigma = Et/[2(1 - \nu)\ r]$, where r is the local radius of curvature[26].

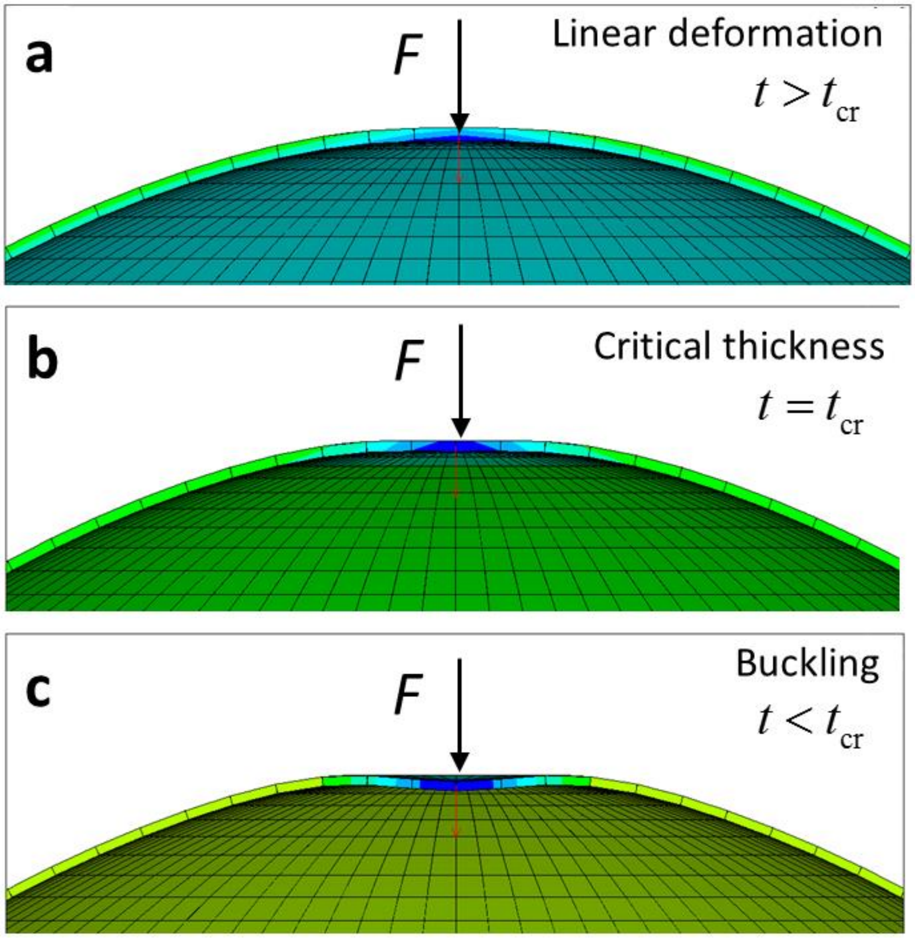

Figure S8. FEM simulations showing buckling failure and critical thickness at buckling. a, Linear regime ($t > t_{cr}$): The displacement is proportional to the force F. **b,** The critical thickness t_{cr} is defined as the thickness at which the curvature is zero within a finite radius from F. **c,** Nonlinear regime ($t < t_{cr}$): The deformed shell exhibits an inverted cap with its maximum displacement proportional to F^2 (ref. [3]).

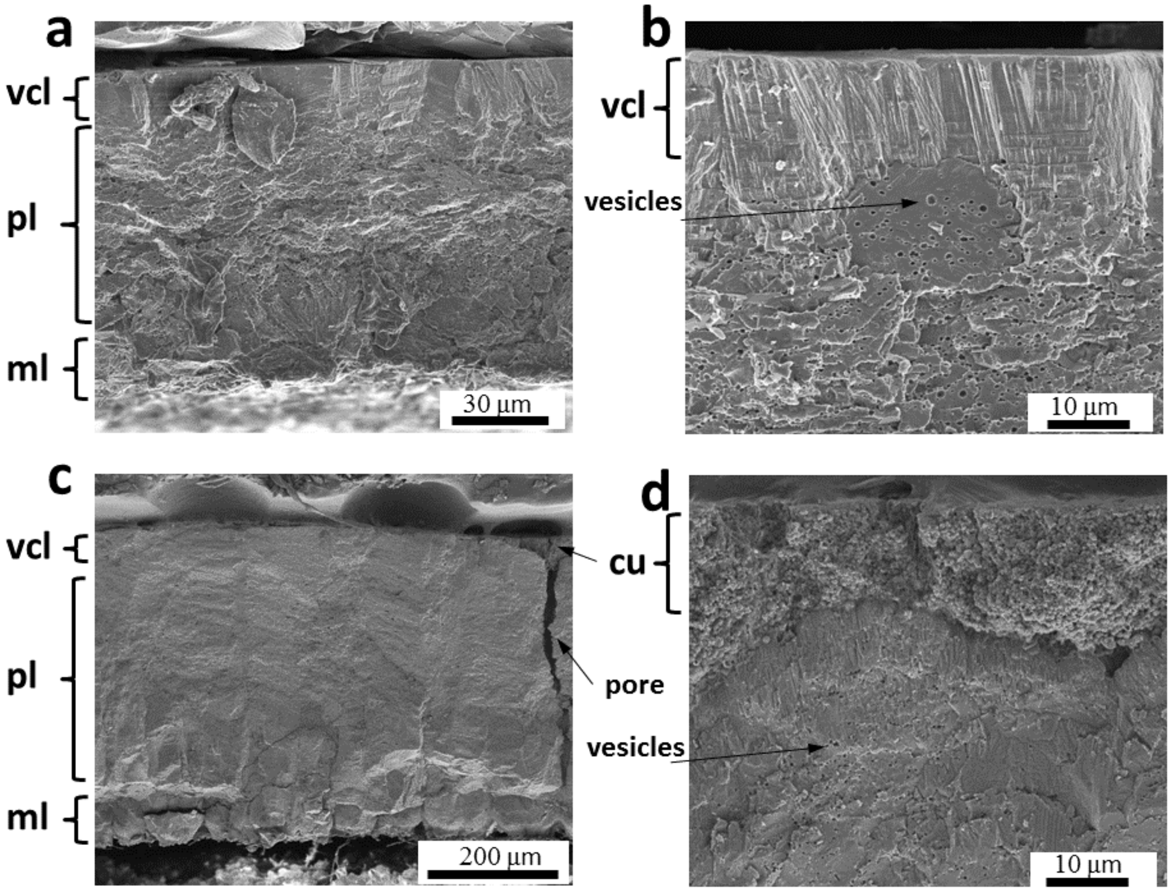

Figure S9. Comparison of eggshell ultrastructure of two representative species. Cross-sectional scanning electron micrographs (SEMs) of **a-b,** Blue-tailed bee-eater, and **c-d,** Indian peafowl (*Pavo cristatus*). The vertical crystal layer has a crystalline structure of higher density than that of the palisade layer. The peafowl eggshell shows much more extensive planes of cleaved calcite than the bee-eater shell, and has smaller and fewer vesicles. The organic constituent is not evenly distributed across the palisade but is more concentrated near the mammillary layer[28]. Abbreviations are as follows: ml: mammillary layer; pl: palisade layer (spongy or squamatic layer[17]); vcl: vertical crystal layer (external zone[17]); cu: cuticle layer.

References

1. Rahn, H., Paganelli, C. V. & Ar, A. Relation of avian egg weight to body weight. *Auk* **92,** 750–765 (1975).
2. Ar, A., Rahn, H. & Paganelli, C. V. The avian egg: mass and strength. *Condor* **81,** 331–337 (1979).
3. Pogorelov, A. V. *Bendings of Surfaces and Stability of Shells*. (American Mathematical Society, 1988).
4. Meyers, M. A., Mckittrick, J. & Chen, P.-Y. Structural Biological Materials: Critical Mechanics-Materials Connections. *Science* **339,** 773–779 (2013).
5. Hauber, M. E., Bates, J. & Becker, B. *The Book of Eggs: A Life-Size Guide to the Eggs of Six Hundred of the World's Bird Species*. (University of Chicago Press, 2014).
6. Meise, W. & Schönwetter, M. *Handbuch der Oologie*. (Akademie Verlag, 1960). doi:10.5962/bhl.title.61353
7. Dunning, J. B. *CRC handbook of avion body masses*. (CRC Press, 2007).
8. del Hoyo, J., Elliott, A., Sargatal, J., Christie, D. A. & de Juana, E. *Handbook of the Birds of the World Alive*. (Lynx Edicions, 2015).
9. Hahn, E. N. *et al.* Natures technical ceramic - the avian eggshell. *J. R. Soc. Interface* **14,** 20160804 (2017).
10. Lazarus, A., Florijn, H. & Reis, P. Geometry-Induced Rigidity in Nonspherical Pressurized Elastic Shells. *Phys. Rev. Lett.* **109,** 1–5 (2012).
11. Nys, Y., Gautron, J., Garcia-Ruiz, J. M. & Hincke, M. T. Avian eggshell mineralization: Biochemical and functional characterization of matrix proteins. *Comptes Rendus - Palevol* **3,** 549–562 (2004).
12. Mikhailov, K. E. Eggshell structure, parataxonomy and phylogenetic analysis: some notes on articles published from 2002 to 2011. *Historical Biology* **0,** 1–11 (2013).
13. Gill, F. B. *Ornithology*. (W. H. Freeman, 2006).
14. Dennis, J. E. *et al.* Microstructure of Matrix and Mineral Components of Eggshells From White Leghorn Chickens (Gallus gallus). *J. Morphol.* **228,** 287–306 (1996).
15. Fecheyr-Lippens, D. C. *et al.* The cuticle modulates ultraviolet reflectance of avian eggshells. *Biol. Open* 1–7 (2015). doi:10.1242/bio.012211
16. Tyler, C. & Simkiss, K. A study of egg shells of ratite birds. *Proc. Zool. Soc. London* **133,** 201–243 (1959).
17. Mikhailov, K. E. Eggshell structure in the Shoebill and pelecaniform birds: comparison with Hamerkop, herons, ibises, and storks. *Can. J. Zool.* **73,** 1754–1770 (1995).
18. Rodriguez-Navarro, A., Kalin, O., Nys, Y. & Garcia-Ruiz, J. M. Influence of the microstructure on the shell strength of eggs laid by hens of different ages. *Br. Poult. Sci.* **43,** 395–403 (2002).

19. Artificially-bred turkey photo. Available at: https://www.hybridturkeys.com/en/.

20. Birkhead, T. *The Most Perfect Thing: Inside (and Outside) a Bird's Egg*. (Bloomsbury USA, 2016).

21. Deeming, D. C. & Ruta, M. Egg shape changes at the theropod – bird transition , and a morphometric study of amniote eggs. *R. Soc. Open Sci.* **1,** (2014).

22. Birkhead, T. R., Thompson, J. E., Jackson, D. & Biggins, J. D. The point of a Guillemot's egg. *Ibis (Lond. 1859).* **159,** 255–265 (2017).

23. Stoddard, M. C. *et al.* Avian egg shape: Form, function, and evolution. *Science* **356,** 1249–1254 (2017).

24. Prum, R. O. *et al.* A comprehensive phylogeny of birds (Aves) using targeted next-generation DNA sequencing. *Nature* **526,** 569–573 (2015).

25. Macleod, N., Bain, M. M. & Hancock, J. W. The mechanics and mechanisms of failure of hens' eggs. *Int. J. Fract.* **142,** 29–41 (2006).

26. Reissner, E. Stresses and displacements of shallow hemispherical shells. *J. Math. Phys.* **25,** 279–300 (1947).

27. Johnson, K. L. *Contact Mechanics*. (Cambridge University Press, 1985).

28. Arias, J. L., Fink, D. J., Xiao, S. Q., Heuer, A. H. & Caplan, A. I. Biomineralization and eggshells: cell-mediated acellular compartments of mineralized extracellular matrix. *Int. Rev. Cytol.* **145,** 217–250 (1993).
